# A thermoresponsive nanocomposite integrates NIR-II-absorbing small molecule with lonidamine for pyroptosis-promoted synergistic immunotherapy

**DOI:** 10.1186/s12951-024-02424-5

**Published:** 2024-04-10

**Authors:** Pengfei Chen, Chi Zhang, Liuliang He, Mingfei Li, Jie Rong, Pengfei Sun, Yingying Chen, Daifeng Li

**Affiliations:** 1 Department of Orthopedics, The First Affiliated Hospital of Zhengzhou University, Zhengzhou, 450052 China; 2 Department of Gynecology, The First Affiliated Hospital of Zhengzhou University, Zhengzhou, 450052 China; 3 State Key Laboratory of Organic Electronics and Information Displays & Institute of Advanced Materials (IAM), Jiangsu Key Laboratory for Biosensors, Nanjing University of Posts & Telecommunications, Nanjing, 210023 China

**Keywords:** Donor engineering, NIR-II absorption, Organic photothermal agents, Pyroptosis, Photothermal immunotherapy

## Abstract

Photothermal immunotherapy is regarded as the ideal cancer therapeutic modality to against malignant solid tumors; however, its therapeutic benefits are often modest and require improvement. In this study, a thermoresponsive nanoparticle (BTN@LND) composed of a photothermal agent (PTA) and pyroptosis inducer (lonidamine) were developed to enhance immunotherapy applications. Specifically, our “two-step” donor engineering strategy produced the strong NIR-II-absorbing organic small-molecule PTA (BTN) that exhibited high NIR-II photothermal performance (ε_1064_ = 1.51 × 10^4^ M^−1^ cm^−1^, *η* = 75.8%), and this facilitates the diagnosis and treatment of deep tumor tissue. Moreover, the fabricated thermally responsive lipid nanoplatform based on BTN efficiently delivered lonidamine to the tumor site and achieved spatiotemporal release triggered by the NIR-II photothermal effect. In vitro and in vivo experiments demonstrated that the NIR-II photothermal therapy (PTT)-mediated on-demand release of cargo effectively faciliated tumor cell pyroptosis, thereby intensifying the immunogenic cell death (ICD) process to promote antitumor immunotherapy. As a result, this intelligent component bearing photothermal and chemotherapy can maximally suppress the growth of tumors, thus providing a promising approach for pyroptosis/NIR-II PTT synergistic therapy against tumors.

**Graphical Abstract:**

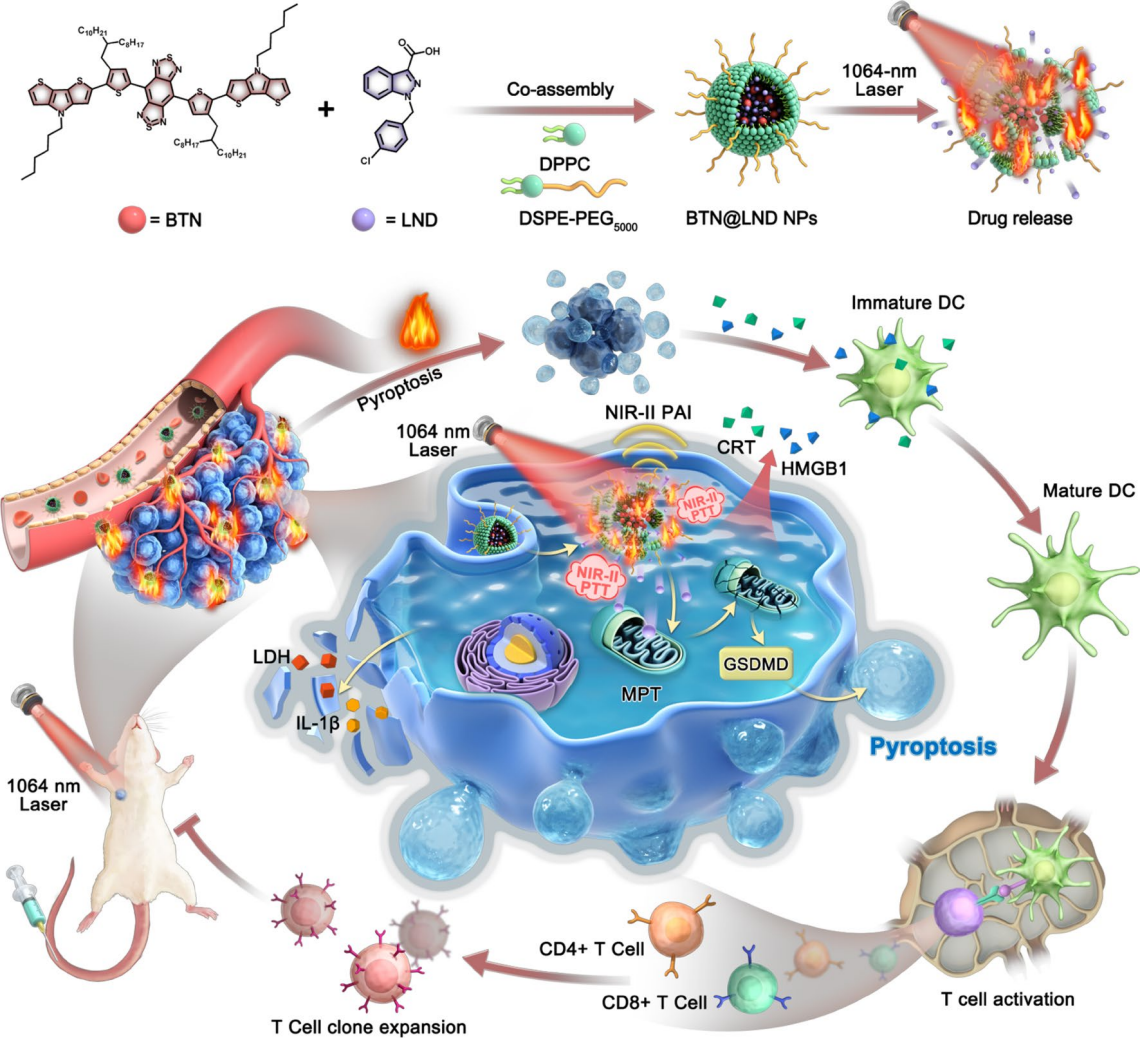

**Supplementary Information:**

The online version contains supplementary material available at 10.1186/s12951-024-02424-5.

## Introduction

The advent of phototheranostics bring new opportunities and great prospects for cancer therapy owing to the non-invasiveness, spatiotemporal control, and negligible side effects [1–5]. As one of the photo-driven therapeutic modalites, photothermal therapy (PTT) involves a process in which the light energy is efficiently converted into local hyperthermia through photothermal agents (PTAs) to causes irreversible damage to intracellular biomolecules [6–9]. As a committed implementor of PTT, PTAs should possess the ability to respondse to the second near-infrared (NIR-II, 1000–1700 nm) laser irradiation, thereby improving tissue penetration depth (~ 10 cm) and tolerance (1064 nm, 1.0 W cm^–2^) [10–14]. Among the various available PTA materials, D–A–D–type organic small molecules have gained considerable attention due to their tunable structure, superior biocompatibility, and great photothermal stability [15–17]. An ideal NIR-II organic small-molecule PTAs should possess both a high NIR-II absorption coefficient (ε) and photothermal conversion efficiency (PCE) that together assure an outstanding photothermal performance (ε × PCE) [18–20]. To attain highly efficient PTAs, several strategies have been commonly used to red-shift the absorption wavelength and boost PCE, including enlarging the π-conjugation extent, matching strong donor–acceptor (D–A) structure, and regulating molecular aggregation [21–23]. However, the structural diversity small-molecule-based NIR-II PTAs remains very limited, and their photothermal performance requires further improvement.

The PTT effect generated by PTAs not only rapidly ablates solid tumors but also induces immunogenic cell death (ICD), and this is promising for cancer immunotherapy [24–30]. However, accumulating evidence has demonstrated that PTA-mediated PTT generally faces the challenge of unsatisfactory immune activation owing to resistance to apoptosis and immunosuppression in tumor cells [31–33]. To activate robust antitumor immune responses, combinations of PTT with chemotherapeutic drugs, gaseous nano-adjuvants, and immunomodulators are highly desirable [9, 34, 35]. Nevertheless, such combined approach-induced hypoimmunogenic apoptotic processes pose a major challenge in regard to achieving antitumor immune activation. Recently, a new paradigm of immunogenic cell death (ICD), termed pyroptosis, was demonstrated to efficiently trigger an acute inflammatory response that produces potent immune activity, and this is promising for antitumor immunotherapy [36–39]. Pyroptosis is mediated by gasdermin (GSDM) family proteins and caspases. [40] The biology of cell pyroptosis is characterized by cell swelling, membrane rupture, and release of cellular contents, including pro-inflammatory cytokines (interleukin-1beta [IL-1β] and interleukin-18 [IL-18]), that can trigger a vigoroso and enduring antitumor immune response [41, 42]. Typically, intracellular GSDMD expression plays a decisive role in apoptosis and pyroptosis after PDT or chemotherapy [43–45]. However, the low expression of GSDMD in most cancer cells tends to limit the occurrence of pyroptotic death after PDT or chemotherapy [46, 47]. The recently discovered mitochondrial permeability transition (MPT), when induced by specific molecular cues, can trigger pyroptosis more rapidly and extensively, and this may enable higher potency for elevating tumor immunogenicity [48]. Therefore, it is highly desirable to utilize the spatio-temporal manipulability of PTT to construct an intelligent nanoplatform for least-harmful precision tumor photo-immunotherapy.

**Scheme 1.**
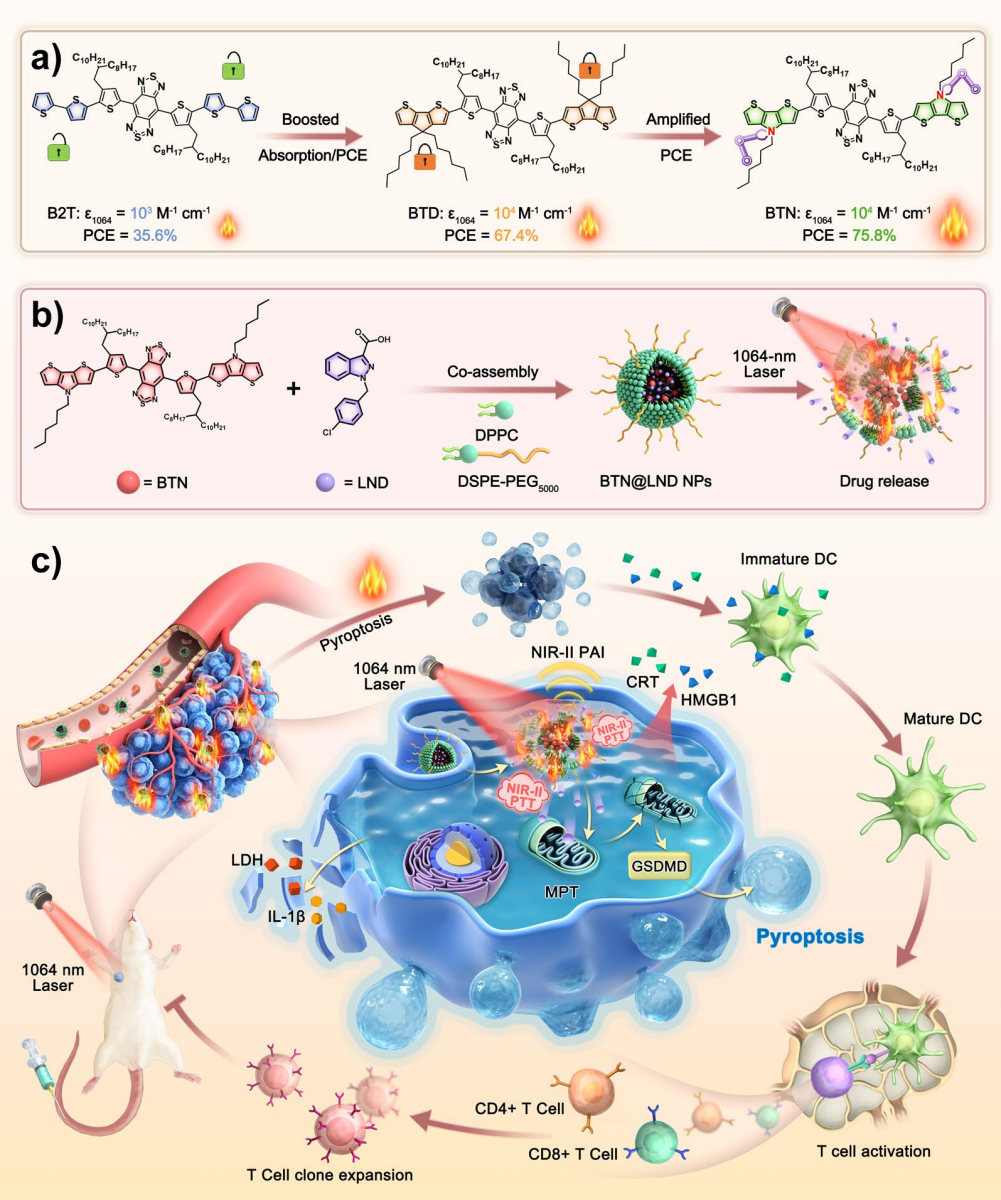
Schematic diagram of NIR-II light-triggered NIR-II PAI guided NIR-II PTT synergia tumor pyroptosis to boost cancer immunotherapy

In this study, we fabricated an intelligent nanoplatform that combined NIR-II PTAs with an MPT inducer for tumor pyroptosis to reinforce antitumor immune responses. First, we employ a “two-step” molecular engineering strategy that aims to generate ideal D–A–D−type PTAs with high NIR-II light trapping capacity and excellent photothermal conversion efficiency (Scheme 1a). The "covalent locking" approach was used to enhance the absorptivity and PCE of PTAs in the NIR-II region by donor unit substitution. The subsequent single-atom substitution (from C to N) method was used to amplify the PCE efficiency of the PTAs. Thus, our donor engineering strategy produced an optimal NIR-II-absorbing PTA (BTN) with high NIR-II photothermal performance (ε_1064_ = 1.51 × 10^4^ M^−1^ cm^−1^, PCE = 75.8%), and this facilitated the diagnosis and treatment of deep tumor tissue. Second, NIR-II PTA (BTN) and an MPT inducer (lonidamine [LND]) were encapsulated in thermally responsive liposomes for delivery to tumor cells (Scheme 1b). Systematic experiments demonstrated that the prepared nanolipid platform not only enables NIR-II PAI-guided NIR-II PTT for the ablation od tumors, but also induces cancer cell pyroptosis by PTT-mediated cargo release that significantly improves the ICD process and activates a robust tumor immune response (Scheme 1c). Compared to the inefficient accumulation and severe side effects caused by free LND when administered systemically that often result in less effective treatments for solid tumors, this NIR-II lipid platform offers a promising approach that integrates precise diagnosis and treatment for improved tumor photothermal immunotherapy.

## Materials and methods

### Preparation of BTN@LND NPs

Referring to previous publications [49], DSPE-PEG (5.0 mg), DPPC (25.0 mg), BTN (1.0 mg) and LND (1.0 mg) were co-dissolved in 5 mL chloroform and the solvent was then evaporated to form a thin film. The obtained thin film was hydrated in 65 °C water under stirring for 10 min, followed by a sonication for another 30 min. After filtration through a 0.22 μm PVDF syringe driven filter, the solution was purified through ultrafiltration to remove unloaded drugs. The obtained BTN@LND was stored at 4 °C for further use. BTN NPs were also prepared using the same method.

### In vitro* NIR-II photothermal release of LND*

In vitro drug release profile of LND were determined by loading 1.0 mL of BTN@LND NPs samples into dialysis tubing (cut-off molecular weight: 100 kDa). The release experiments were performed using 1064 nm NIR-II laser (1.0 W/cm^−2^) at indicated time points. At predetermined time points, the daily sate was taken out to estimate the amount of drug released. The LND concentration in the samples was measured by absorption spectroscopy.

### In vitro* cellular uptake*

First, the 4T1 cells seeded 1 × 10^5^ per well and condition for 12 h. Then, adding the FITC(BTN@LND) NPs (0.1 mg/mL) to incubate with 4T1 cells for another 8 h. Afterward, the cells were washed with PBS for three times, followed by costaining with Lyso-Tracker Red (lysosome indicator, 30 min, KeyGen Biotech.) plus Hoechst 33342 (nucleus indicator, 10 min, KeyGen Biotech.). After washing the samples with PBS, the fluorescence images were captured by confocal fluorescence microscope. For the flow cytometry detection, the upper supernatant was sucked out and 1.0 mL of PBS was added to clean twice and remove dead cells, and preparation of cell suspension.

### In vitro* cytotoxicity assay*

The 4T1 cells (1 × 10^5^ cells/well) were incubated in a 96-well plate (KeyGen Biotech.) for 12 h. Materials in different groups (PBS, BTN@LND, Free LND, BTN + L, and BTN@LND + L) were added to fresh DMEM to obtain the different mixed medium (0, 0.0125, 0.025, 0.05, 0.1, and 0.2 mg mL^−1^). After further incubation for 12 h, the cells were washed for three times. Thereafter, selected wells from the different groups were illuminated with or without laser irradiation at 1064 nm (1.0 W cm^−2^ for 5 min). After another incubation for 24 h, each well of the microliter plate was added MTT solution and the plate was cultured for accessional 3 h. Finally, the Bio-tek Synergy HTX microplate spectrophotometer was used to determine the 490 nm absorbance of each well.

### Detection of immunogenic cell death (ICD) biomarkers

The 4T1 cells (1 × 10^4^) were grown on slices in a 12-well culture plate for 12 h. Materials in different groups (PBS, BTN@LND, Free LND, BTN + L, and BTN@LND + L) were added to fresh DMEM to obtain the mixed medium (0.1 mg/mL) for 12 h with or without laser irradiation at 1064 nm (1.0 W cm^−2^ for 5 min). Then, the 4T1 cells were fixed in 4% paraformaldehyde for 30 min, and incubated at 4 ℃ overnight with CRT or HMGB-1 primary antibody. Goat Alexa Fluor 488 anti-rabbit IgG secondary antibody for 2 h at room temperature. Finally, the 4T1 cells were stained with DAPI for another 5 min. After washing with the PBS, the fluorescence images of samples were captured by confocal fluorescence microscope.

### In vivo* immunofluorescence assay*

To systemically evaluate the immune response, 4T1 tumor-bearing mice were euthanized after different treatments, and tumor draining lymph nodes, spleens, and tumors were harvested and prepared into single-cell suspensions according to the manufacturer’s protocols, respectively. The collected single-cell suspensions were blocked with anti-mouse CD16/32 for 10 min to avoid nonspecific binding. The fixable viability stain 780 was used to distinguish between living and dead cells, and then stained with various fluorescence-labeled antibodies according to the manufacturer’s instructions (BD Pharmingen). The following antibodies were used in the analysis of DCs: anti-CD45-FITC, anti-CD11c-PE, anti-CD80-APC, and anti-CD86-PE-Cy7. The following antibodies were used in the analysis of CTLs, the antibodies were as follows: anti-CD45-FITC, anti-CD3-APC, anti-CD4-PE, and anti-CD8-PerCP-Cy5.5. All antibodies were stained for 30 min, followed by washing three times with cell staining buffer. The immunocellular percentages were analyzed by flow cytometry. Blood samples in each group were collected for the detection of TNF-α and IL-6 by ELISA according to the manufacturer’s protocols.

## Results and discussion

### Molecular design and theoretical calculations

**Fig. 1:**
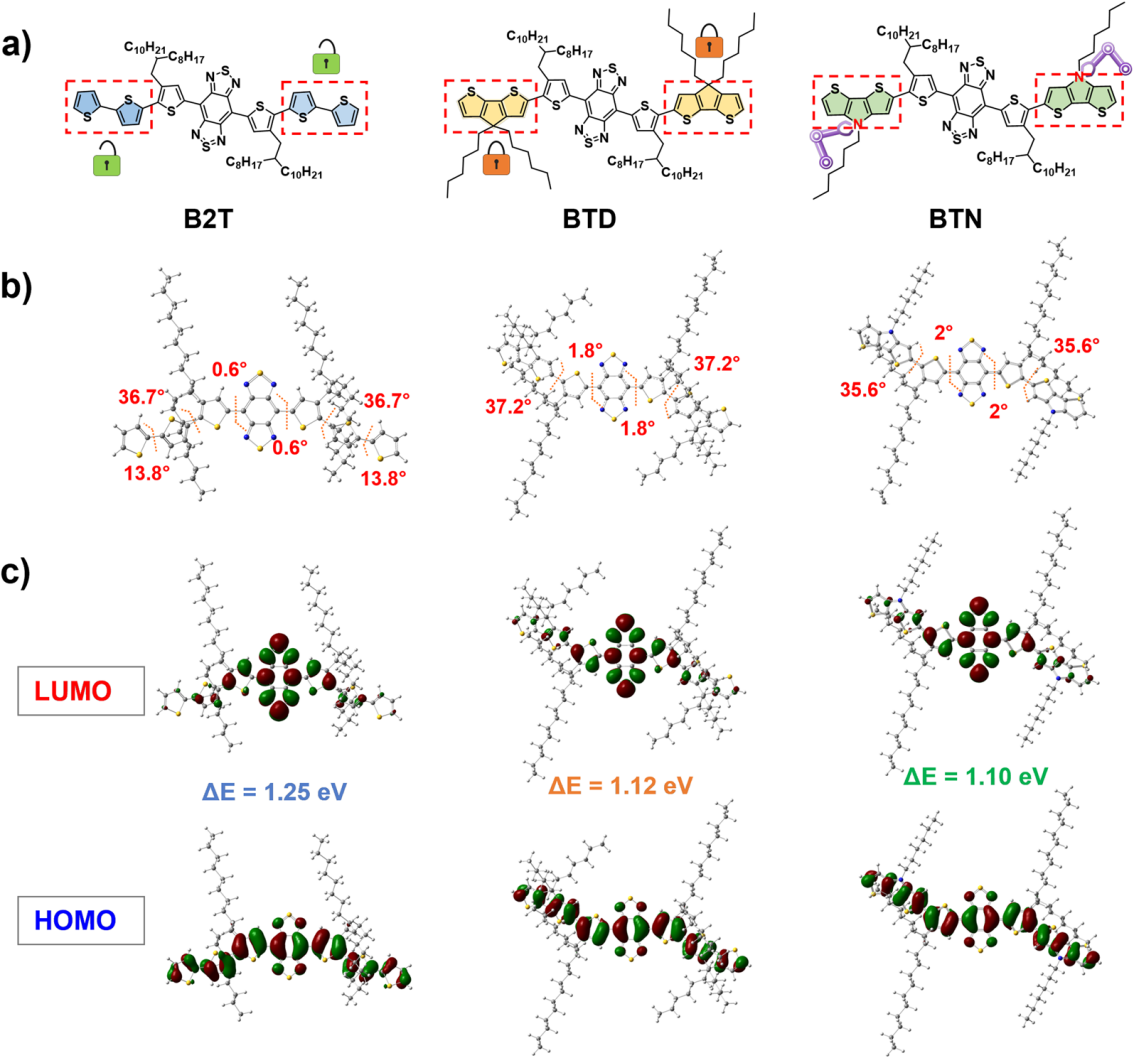
**a** Chemical structures of B2T, BTD, and BTN. **b** Optimized geometries and **c** HOMO/LUMO levels

The ingenious integration of intense D‒A strength with planar conjugate skeletons has been proven to be an attractive approach for the development of PTAs with long absorption and high photothermal performance (ε × PCE). Our recent research demonstrated that exploiting the strategy of intramolecular conformational locks to control the planarity of donor units bearing 2,2′-bithiophene (2T) derivatives can significantly amplify the absorptivity (ε) and PCE of PTAs [50]. However, this strategy is limited by the strength of the weak intramolecular interactions (S–O), ultimately resulting in an extremely restricted effect. In this study, we aimed to create a more planar donor unit (cyclopenta[1,2-b:5,4-b′]dithiophene [CPDT]) by locking the two thiophenes into a conjugated plane through the insertion of covalent bonds containing an sp^3^-hybridized carbon (C) atom (Fig. 1a). This chemical modification would produce a richer delocalized charge density than that of the 2 T group. Hence, the introduction of a CPDT unit into molecular structures may be favorable for reducing the band gap and improving the overall molecular planarity. We took advantage of the CPDT derivative as the donor unit in the small-molecule backbone and integration of benzo[1,2-′c:4,5-c′]bis[1,2,5]thia-diazole (BBTD) electron-accepting segment to elevate the ε and PCE in the NIR-II region. Furthermore, aiming at obtaining superior photothermal performances, substitution of the sp^3^-hybridized nitrogen (N) in place of C in the donor was conducted in the molecule design in view of the presence of p–π conjugated and a stronger surface electrostatic potential. Moreover, to improve the solubility and minimize the impact on the backbone structure of the PTAs, we manipulated the location of the alkyl chain on the BBTDT moiety. The detailed syntheses are presented in Additional file 1: Schemes S1–S3, and they were characterized using nuclear magnetic resonance (NMR) and mass spectrometry (Additional file 1: Figs. S1–S9)

To verify the viability of the molecular design, density functional theory at the B3LYP/6-31 g* level was used to decipher the geometric and electronic properties at the molecular level in the ground state (S_0_). As presented in Fig. 1b, the optimized S_0_ geometries of B2T, BTD, and BTN indicate that the dihedral angle between the central BBT acceptor and the nearby thiophene unit is less than 2°, thus suggesting that the BBTDT core is a planar acceptor. The total dihedral angle between the BBTDT core and the 2 T donor was 50.5° in B2T, indicating that the relatively distorted molecular architecture was primarily caused by steric hindrance between the thiophene rings with single bonds in series. When the two thiophene rings in B2T were replaced by a more planar cyclic fluorene/pyrrole ring, the dihedral angles between BBTDT and the donor ring decreased to 37.2° and 35.6°, respectively. Meanwhile, the overall planarity of the molecular backbone was much higher than that of B2T, as the second angle of the donor disappeared. The highest occupied molecular orbitals (HOMOs) of B2T, BTD, and BTN were off-domain along the entire conjugated backbone, whereas the lowest unoccupied molecular orbitals (LUMOs) were predominantly distributed in the BBTDT core (Fig. 1c), suggesting the presence of a significant ICT effect. Correspondingly, the calculated energy band gaps of B2T, BTD, and BTN gradually decreased from 1.25 and 1.12 to 1.10 eV, thus demonstrating their increasing electron-donating ability and ICT effect.

### Photophysical and photothermal properties

**Fig. 2:**
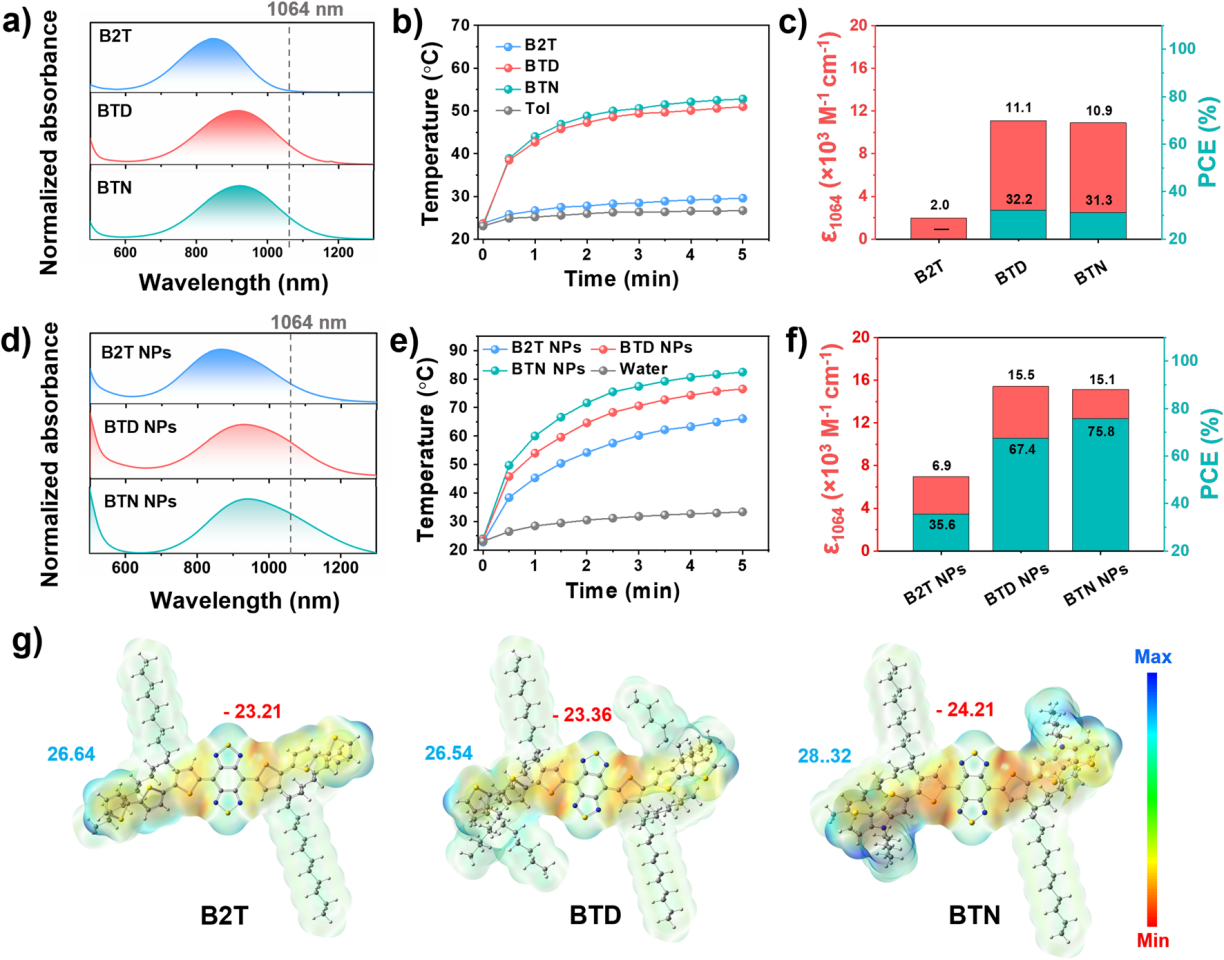
**a** Normalized absorption spectra of monomer in THF. **b** Photothermal curves in toluene under laser (1064 nm, 0.60 W cm^−2^) irradiation (concentration: 0.1 mg mL^−1^). **c** Molar extinction coefficients at 1064 nm (red) and the PCE (cyan) in toluene. **d** Normalized absorption spectra of NPs in aqueous solution. **e** Photothermal curves in water under laser (1064 nm, 1.0 W cm^−2^) irradiation (concentration: 0.1 mg mL^−1^). **f** Molar extinction coefficients at 1064 nm (red) and the PCEs (cyan) in water. **g** Maps of electrostatic potential (ESP) surfaces for the compounds

The photophysical properties of B2T, BTD, and BTN were characterized using UV–visible-NIR spectroscopy. As presented in Fig. 2a, all exhibit broad absorption in THF solution with maximum peaks at 858, 915, and 923 nm, and this is consistent with the theoretically calculated band gap. As expected, the "covalent locking" strategy drives the expansion of their absorption peaks into the NIR-II region. Moreover, the molar extinction coefficients (ε) of B2T, BTD, and BTN at the 1064 absorption peak were 1979, 11,095, and 10,944 L mol^−1^ cm^−1^, respectively (Fig. 2c and Additional file 1: Fig. S10). It is observed that the ε of BTD and BTN at 1064 nm are approximately 5.5-fold higher than that of B2T, indicating that the introduction of large planar and electron-rich donor untis can significantly improve the NIR-II light-harvesting ability. Subsequently, the photothermal effects of B2T, BTD, and BTN in toluene solution (0.1 mg mL^−1^) were evaluated under 808 nm and 1064 nm laser irradiation (0.6 W cm^−2^), respectively. As illustrated in Additional file 1: Fig. S11, under 808 nm laser irradiation, their heating curves were similar, and this was attributed to their favorable light-absorbing ability at this wavelength. In contrast, steep heating curves were observed in the BTD and BTN solutions under 1064 nm laser irradiation, whereas the heating curve for B2T was relatively flat. This resulted from the larger absorption capacities of BTD and BTN. After 5 min of continuous 1064 nm laser irradiation (0.60 W cm^−2^), the temperatures of BTD and BTN solutions (0.1 mg mL^−1^) reached plateau temperatures of 50.9 °C and 51.7 °C, respectively (Fig. 2b). Moreover, the photothermal conversion efficiencies (PCE) of BTD and BTN nanoparticles (NPs) were determined to be 32.2 and 31.3%, respectively (Fig. 2c). Consequently, these results confirmed that our "covalent locking" approach can generate superior NIR-II PTAs with high NIR-II absorptivity.

To further evaluate the photophysical properties of the aqueous solution, the molecules were transformed into water-soluble NPs through an ultrasound-assisted nanoprecipitation strategy with DSPE-PEG_5000_. Compared to their THF solutions, the UV–Vis-NIR absorption of the three NPs was slightly red-shifted to 874, 931, and 940 nm (Fig. 2d). Remarkably, the tail peaks were significantly elevated due to the formation of aggregates, ultimately resulting in a significant enhancement of their ε_1064_ calculated as 6977, 15427, and 15120 L mol^−1^ cm^−1^, respectively (Fig. 2f and Additional file 1: Fig. S12). Considering their outstanding 1064 nm absorptivity in water, the light-to-heat ability was further investigated by observing the temperature changes at different points in time. As depicted in Fig. 2e, after laser (1064 nm, 1.0 W cm^−2^) irradiation for 5 min, the temperature of BTD and BTN NPs (0.1 mg mL^−1^) dramatically increased from ~ 25 to ~ 80°C, and this was greater than that of B2T NPs (66°C). The PCE of B2T, BTD, and BTN NPs were determined to be 35.6%, 67.4%, and 75.8%, respectively (Fig. 2f and Additional file 1: Fig. S13), in which of the BTN NPs exceeded most of the currently reported NIR-II PTAs bearing D–A–D–type small molecules. Based on the reported formulas, the photothermal performances of B2T, BTD, and BTN were calculated as 2.46 × 10^3^, 1.05 × 10^4^, and 1.15 × 10^4^, respectively. Collectively, our “two-step” donor engineering strategy successfully generated an ideal D–A–D–type PTA (BTN) with high NIR-II light trapping capacity and excellent photothermal conversion efficiency.

Notably, there was no significant difference in the PEC performance between BTD and BTN in the monomeric state; however, the gap in PEC performance in the NPs state was amplified. This may be related to their aggregated states [15, 51, 52]. To understand this, the surface electrostatic potentials (ESP) of these molecules were calculated separately based on the Gaussian optimized structures. As shown in Fig. 2g, the negative electrostatic potential (red) is located on the thiophene bridges on both sides of the BBTDT core, whereas the positive electrostatic potential (blue) is primarily observed at the ends of the donor rings. A prominent ESP difference value was observed between donor and acceptor units. For BTN the value is 52.52 kcal/mol, and this is much larger than those of B2T and BTD (49.85 and 49.90 kcal/mol^−1^). A larger ESP difference value induces the BTN to form charge-transfer states and stronger D–A interactions between neighboring molecules, thereby favoring the generation of nonradiative-leap channels [15, 17, 53–55]. Overall, the BTN NPs were selected for subsequent studies due to their outstanding NIR-II absorption and photothermal performance.

### Preparation and characterization of BTN@LND NPs

**Fig. 3:**
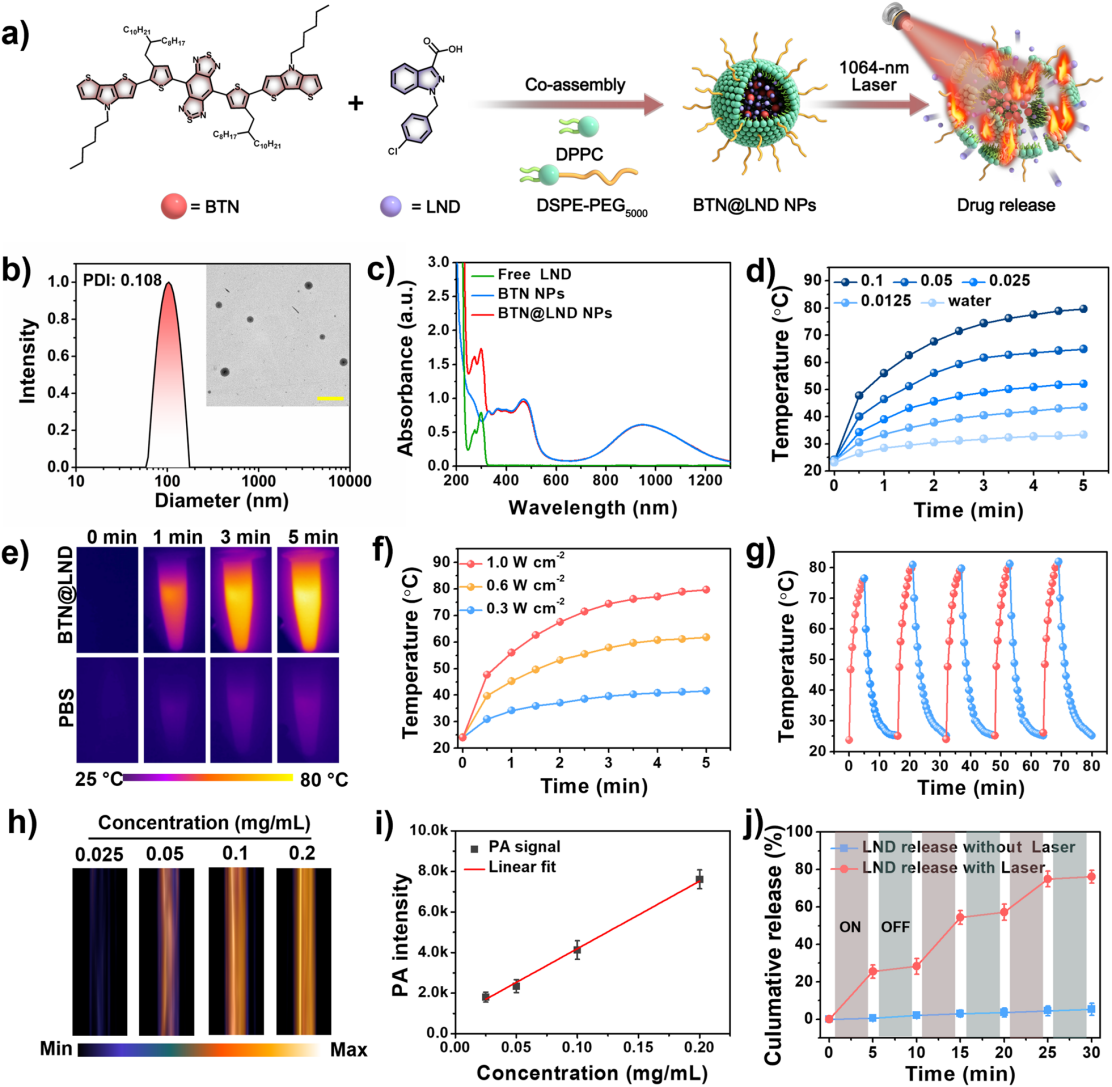
**a** The preparation of BTN@LND NPs and NIR-II PTT-triggered drug release. **b** DLS and transmission electron microscopy (TEM) image of BTN@LND NPs. **c** UV–Vis-NIR absorption spectra of free LND, BTN NPs, and BTN@LND NPs. **d** Concentration-dependent photothermal curves of BTN@LND NPs (1064 nm laser, 1.0 W cm^−2^). **e** Time-dependent infrared thermographs of BTN@LND NPs (0.1 mg mL^−1^, 1064 nm laser, 1.0 W cm^−2^). **f** Photothermal heating curves of BTN NPs with different power densities for comparison. **g** Five heating and cooling cycles of BTN NPs (0.1 mg mL^−1^, 1064 nm laser, 1.0 W cm^−2^). **h** Relevant in vitro PA images with different concentration. **i** Concentration-dependent PA intensities of BTN@LND NPs. Inset: relevant in vitro PA images. **j** NIR-II PTT-triggered LND release curves from BTN@LND NPs. Means ± SD, n = 3

Based on the ideal photothermal properties of BTN NPs, we considered the construction of a thermally responsive lipid nanodrug delivery system for NIR-II PTT/chemotherapy synergistic therapy with 1064 nm light triggering the release of lonidamine (LND). BTN@LND NPs were fabricated according to existing methods (Fig. 3a). The BTN@LND NPs exhibited a spherical morphology with a hydrodynamic diameter of approximately 100 nm (Fig. 3b). The dynamic light scattering (DLS) results of BTN@LND NPs remained essentially unchanged for a sustained period of 7 days in PBS buffer, dulbecco's modified Eagle’s medium (DMEM), and 10% fetal bovine serum (FBS), thus demonstrating their high stability (Additional file 1: Fig. S14). From the absorption spectra, we confirmed that LDN was successfully loaded into the NPs, and the encapsulation efficiency was determined to be 33%. This is attributed to the combined dominance of hydrophobic and π–π interactions (Fig. 3c). More significantly, LND loading exerted a negligible effect on the photothermal performance of the NPs (Additional file 1: Fig. S15). Moreover, the temperature variation of the BTN@LND NPs was positively correlated with the laser power density and concentration (Fig. 3d, f), thus indicating controllable photoheating behavior. BTN@LND NPs can be recognized by photothermal imaging (PTI) of the thermal signals generated due to their good photothermal effect, and Fig. 3e presents its time-dependent temperature process. Five laser on/off heating cycles demonstrated the outstanding photothermal stability of BTN@LND NPs (Fig. 3g). Meanwhile, the non-radiative processes generated by the nanoparticles under 1064 nm laser irradiation also triggered photoacoustic (PA) signals, and the intensity was positively correlated with the concentration (15– 120 μM, Fig. 3h, i). The significant PA signal lays a solid foundation for direct PTT of the BTNA@LND NPs via NIR-II PAI. Finally, the NIR-II photo-triggered LND release from BTN@LND NPs was investigated under 1064 nm laser irradiation. Under NIR-II laser irradiation, localized heat induced a phase transition of the complex phospholipids, and the drug was rapidly released from the molten liposomes. As presented in Fig. 3j, the release behavior of LND under 1064 nm laser irradiation (1.0 W cm^−2^) exhibited an obvious “on–off” release characteristic. In contrast, no significant release was observed in the control group without 1064 nm laser irradiation, thus suggesting that the release of LND was triggered only by the photothermal effect of BTN induced by NIR-II light.

### Evaluation of anticancer effect and pyroptosis-induced ability

**Fig. 4:**
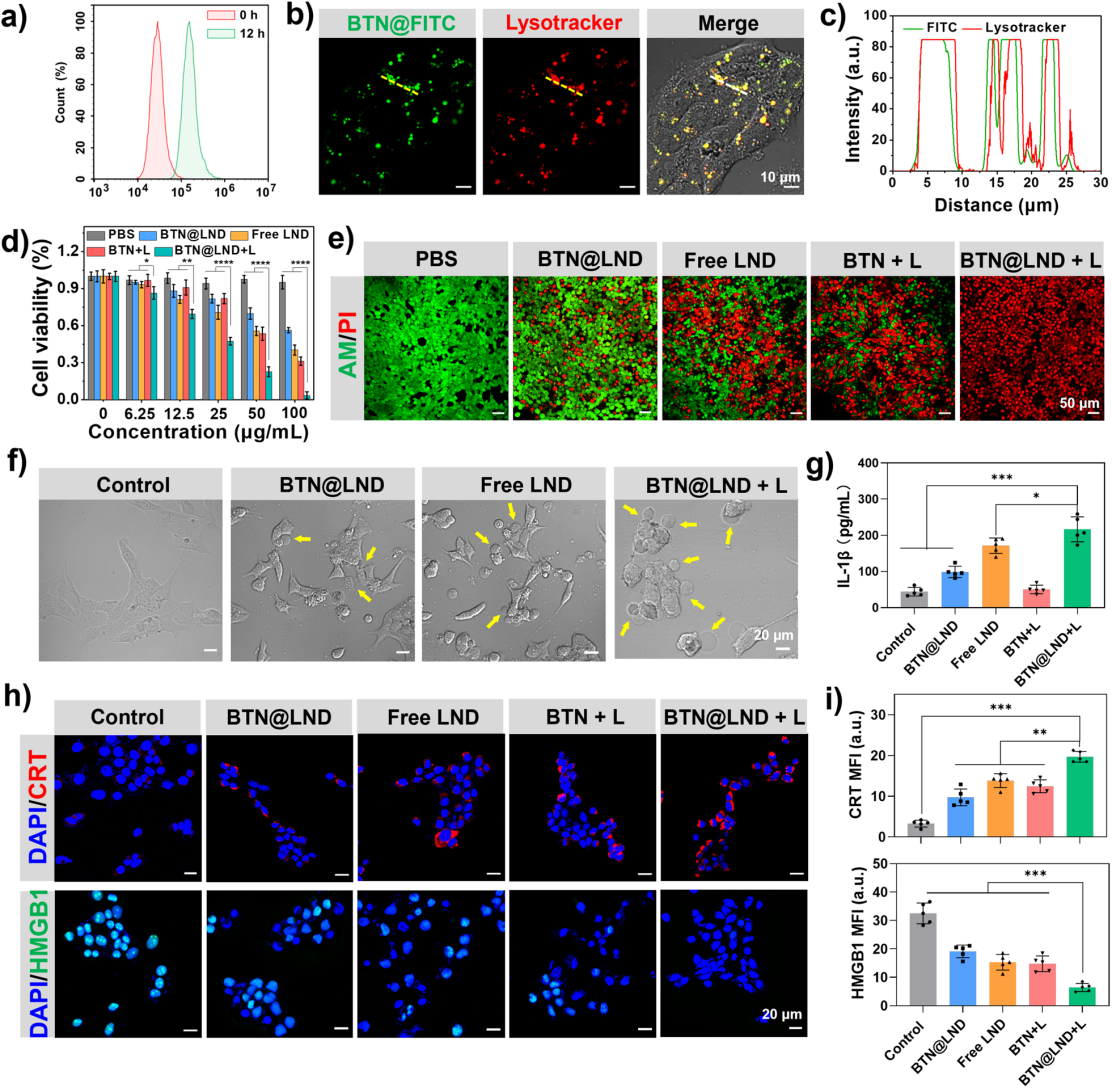
**a **The cell uptake of FITC@BTN NPs incubated with 4T1 cells for 12 h by flow cytometry monitor. **b** Colocalization fluorescence imaging of 4T1 cells after incubation with FITC@BTN NPs (0.1 mg mL^−1^) for 12 h. **c** Fluorescence overlap analysis of the dashed line segment in b) (Green channel: FITC@BTN NPs, Red channel: Lysotracter). **d** Relative viabilities of 4T1 cells after different treatments (1064-nm laser: 1.0 W cm^−2^, 5 min) (n = 5). **e** Live/dead co-staining images of 4T1 cells with different treatments (1064-nm laser: 1.0 W cm^−2^, 5 min). **f** Representative bright-field microscopy image of 4T1 cells treated with different formulations. yellow arrows indicated the pyroptotic cells. **g** ELISA assay for IL-1β secretion in 4T1 cell supernatant receiving different treatments (n = 5). **h** CLSM images of 4T1 cells for CRT and HMGB-1 assay in different groups. **i** Quantification of CRT and HMGB-1 expression levels after different treatments (n = 5). *P < 0.05, **P < 0.01, ***P < 0.001, ***P < 0.0001

Prior to the evaluation of phototheranostics, the cellular uptake of BTN@LND NPs was assessed using confocal fluorescence microscopy (CLSM). Considering the non-emitting characteristics of the BTN@LND NPs, a commercial green fluorophore (FITC) was encapsulated in the BTN@LND NPs to observe their fate in 4T1 cells. As presented in Additional file 1: Fig. S16, strong green fluorescent signals were observed in 4T1 cells after incubation with FITC/BTN@LND NPs for 12 h. Flow cytometric analysis revealed strong and similar fluorescence signals in 4T1 cells after incubation with FITC/BTN@LND NPs for 12 h (Fig. 4a). These results indicate successful cellular uptake. The co-localization experiment further demonstrated that the green fluorescence signals of FITC/BTN@LND NPs were highly coincident with LysoTracker Red (Fig. 4b, c), thus indicating that the nanoparticles were transported into the lysosomal organelles through the endocytosis pathway [56].

Based on the efficient cellular uptake, the in vitro phototheranostic effects of BTN@LND NPs on 4T1 cells were assessed using the 3-(4,5-dimethylthiazol-2-yl)-2,5-diphenyl tetrazolium bromide (MTT) assay. In the dark, BTN@LND NPs exhibited negligible cytotoxicity even at concentrations of 0.1 mg mL^−1^, suggesting a good biocompatibility (Fig. 4d). In contrast, the BTN@LND, free LND, and BTN + L groups exhibited concentration-dependent inhibition of cellular activity with 56%, 40%, and 31% inhibition, respectively. Remarkably, the BTN@LND + L group caused death to over 95% of 4T1 cells after 1064 nm laser irradiation (1.0 W cm^−2^) due to the advantages of combination therapy. To visually highlight the phototherapeutic effect of the BTN@LND NPs, a live-dead staining method (calcein-AM/propidium iodide) was used to distinguish between live (green) and dead (red) 4T1 cells. As presented in Fig. 4e, only green fluorescence was observed in the PBS group, whereas mixed red and green signals were observed in the BTN@LND, free LND, and BTN + L groups, thus suggesting that the dark toxicity of the drug and the phototoxicity of BTN acted separately. Notably, a large amount of red fluorescence was predominant in the BTN@LND + L group, suggesting a robust tumor cell ablation capability. These results demonstrate the remarkable in vitro synergistic effect of NIR-II PTT and LND drugs for enhancing their anticancer effects.

Subsequently, the death patterns were further evaluated by observing 4T1 cell morphology. The BTN@LND and free LND groups exhibited a typical pyroptotic morphology with cell swelling and membrane blister formation (yellow arrows) after co-incubation for 4h (Fig. 4f). Interestingly, pyroptosis was induced in numerous cells in the BTN@LND + L group compared to that in the other treatments, and this could be attributed to the rapid intracellular release of PTT-induced drugs. As significant signs of intracellular contents leakage, the release of lactate dehydrogenase (LDH) and inflammatory cytokine (IL-1β) was detected during focal death (Fig. 4g and Additional file 1: Fig. S17). Based on the above results, LND-loaded nanomedicines exhibited moderate therapeutic efficacy that may be attributed to the difficulty in achieving rapid release of the drug from the nanosystem under dark conditions to enhance local drug concentration, thus generating a molecular message to activate pyroptosis. Notably, compared to that of the free LND group, the superior pyroptosis effect provided by BTN@LND NPs + 1064 nm laser irradiation may be attributed to NIR-II PTT triggering, ultimately resulting in localized production of high drug concentrations. Collectively, these results strongly suggest the significance of NIR-II PTT-controlled release of LND in tumor cell pyroptosis.

It has been demonstrated that cell pyroptosis is a potent immunogenic cell death (ICD) form. Therefore, the pyroptosis-mediated ICD process was further assessed by immunofluorescence detecting two important markers that included calreticulin (CRT) and high mobility group box-1 (HMGB-1). As presented in Fig. 4h, BTN@LND-treated 4T1 cells significantly promoted CRT exposure on the cell surface under 1064 nm laser irradiation. Moreover, HMGB-1 was initially detected in the nucleus but migrated out of the nucleus almost entirely after receiving BTN@LND + 1064 nm laser treatment. Furthermore, relative mean fluorescence intensity (MFIs) analysis demonstrated that the BTN@LND group receiving 1064 nm laser irradiation exhibited the highest release of DAMPs (Fig. 4i). These results clearly indicate that the level of DAMPs release was more significant in the BTN@LND + L group than it was in the free LND and BTN + L groups, thus suggesting that the cellular focalization induced by the BTN@LND + L group after NIR-II light irradiation exhibited better immunogenicity.

### NIR-II light-triggered antitumor efficacy in vivo

**Fig. 5:**
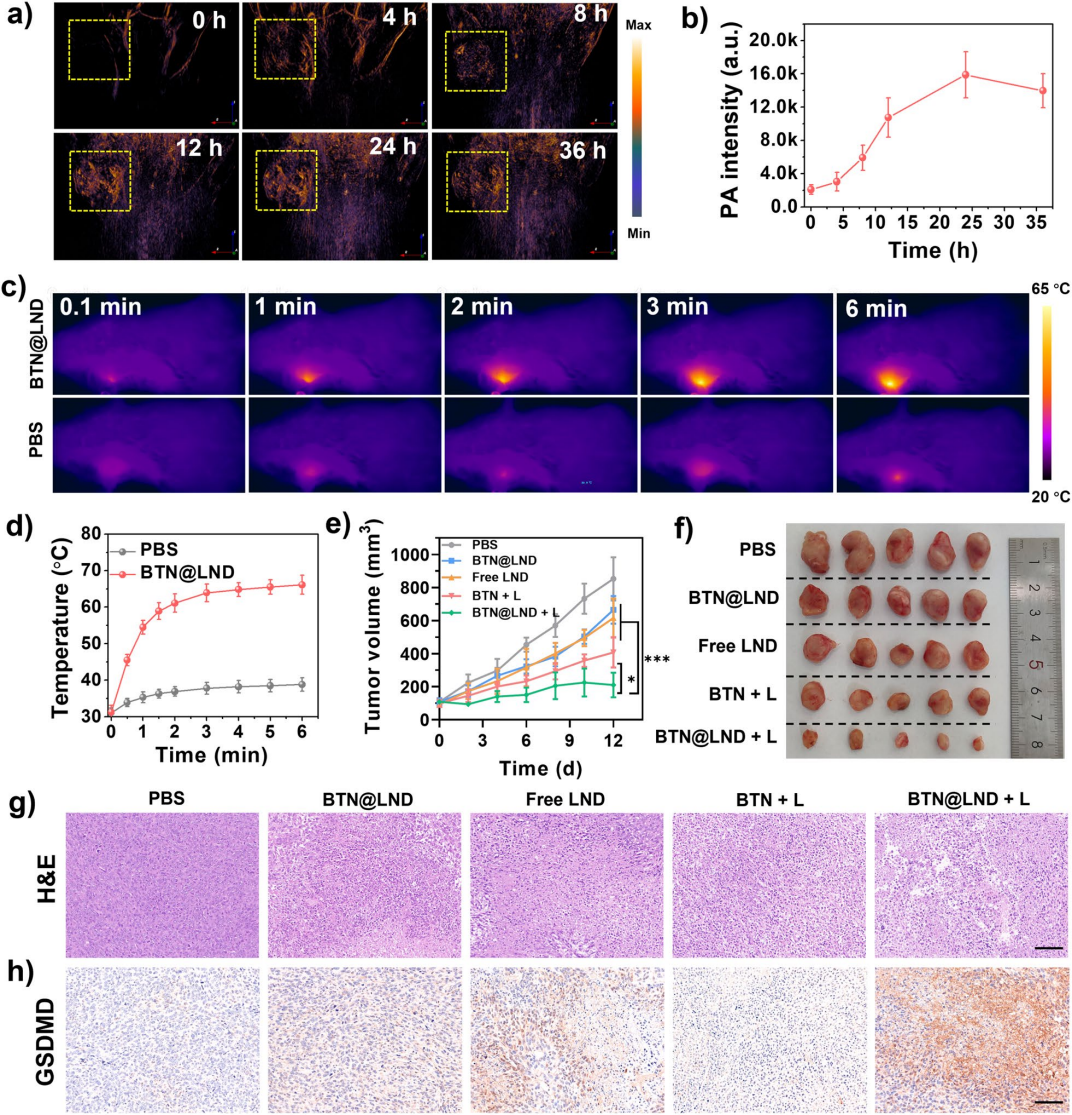
**a** NIR-II PAI of BTN@LND NPs in vivo under 1064-nm pulsed laser excitation. **b** PA intensity in the tumor site (n = 3). **c** Infrared thermograms of the tumor site at different time points under 1064-nm laser irradiation (1.0 W cm^−2^). **d** Heating curves in the tumor site (n = 3). **e** Tumor growth curves after receiving different treatments (n = 5). **f** Final ex vivo tumor images after receiving different treatments. **g** Histological examination of 4T1 tumors in different groups, scale bar = 100 μm. **h** Expression levels of GSDMD in different treatments, scale bar = 100 μm. *P < 0.05

These encouraging in vitro results prompted us to investigate the potential of BTN@LND NPs for in vivo imaging-guided PTT. To detect the biodistribution of NPs in vivo, the real-time NIR-II PAI were accessed via injection of BTN@LND NPs (150 μL, 2.0 mg mL^−1^) into BALB/c mice with 4T1 subcutaneous tumors. As presented in Fig. 5a, b, the NIR-II PAI signal gradually increased with time in the tumor section and reached a maximum level at 24 h, thus highlighting the prominent tumor-seeking ability of BTN@LND NPs. After 36 h of injection, the PA signal exhibited slight weakening. Therefore, the best time of laser irradiation was determined to be 24 h after the injection. Meanwhile, as demonstrated in ex vivo investigation, the PA signal in the tumor region was significant, indicating successful accumulation. Due to their metabolic action, BTN@LND NPs also exhibited higher accumulation in the liver and spleen but lower deposition in the heart and kidneys (Additional file 1: Fig. S18). Additionally, the photothermal performance of the BTN@LND NPs was further evaluated by infrared thermal imaging at 24 h after tumor injection (Fig. 5c). As shown in Fig. 5d, the temperature of the tumor site rose sharply to 55 °C within 1 min and remained at approximately 63 °C for the remainder of the experiment under 1064 nm laser irradiation (1.0 W cm^−2^). In contrast, the PBS + 1064 nm laser displayed only a slight increase in temperature under this condition.

Evaluation of the in vivo anti-tumor capacity of BTN@LND NPs guided by dual-modality imaging (NIR-II PAI/PTI) after confirming their retention time and heating profile in tumor tissues was performed. A unilateral 4T1 subcutaneous tumor model was established and randomly assigned into five groups (PBS, BTN@LND, Free LND, BTN + L, and BTN@LND + L) that received different treatments. At 24 h postinjection, tumor sites in the BTN + L and BTN@LND + L groups were exposed to laser (1064 nm, 1.0 W cm^−2^) irradiation for 6 min. As presented in Fig. 5e, after 12 days of treatment, the anti-tumor efficacy of the BTN@LND and free LND groups was very weak (6.2-fold and 6.6-fold increases in 4T1 tumor volume, respectively) than that of the PBS group (7.9-fold increase in 4T1 tumor volume). The BTN + L group exhibited a 4.1-fold increase in 4T1 tumor volume, thus suggesting that a single NIR-II PTT treatment exerted a better inhibitory effect on tumor growth than did the BTN@LND and free LND groups. In sharp contrast, the BTN@LND + L group exhibited optimal tumor suppression on account of the cooperation of NIR-II PTT and chemotherapy (a 1.9-fold increase in tumor volume). Moreover, photographs of each group of tumors in isolation reflected this observation (Fig. 5f). Additionally, the body weights of the mice in all groups were maintained within a reasonable range during the treatment period (Additional file 1: Fig. S19). Hematoxylin and eosin (H&E) staining of tumor sections after different treatments clearly indicated that severe damage to tumor tissues occurred in the BTN@LND + L group due to the synergistic effects of NIR-II PTT and chemotherapy (Fig. 5g). Immunohistochemical analysis of the tumor sites after different treatments clearly demonstrated that GSDMD exhibited the highest expression level in the BTN@LND + L group, and this could be attributed to the accurate on-demand release of the LND pyroptosis inducer via NIR-II PTT (Fig. 5h). Additionally, the biosafety and biocompatibility of BTN@LND NPs were evaluated during the treatment period. The state of each organ was determined using H&E staining. No pathological abnormalities were observed in the major tissues of the treatment groups compared to the controls (Additional file 1: Fig. S20), thus indicating minimal side effects of BTN@LND NPs.

### *Antitumor immune response *in vivo

**Fig. 6:**
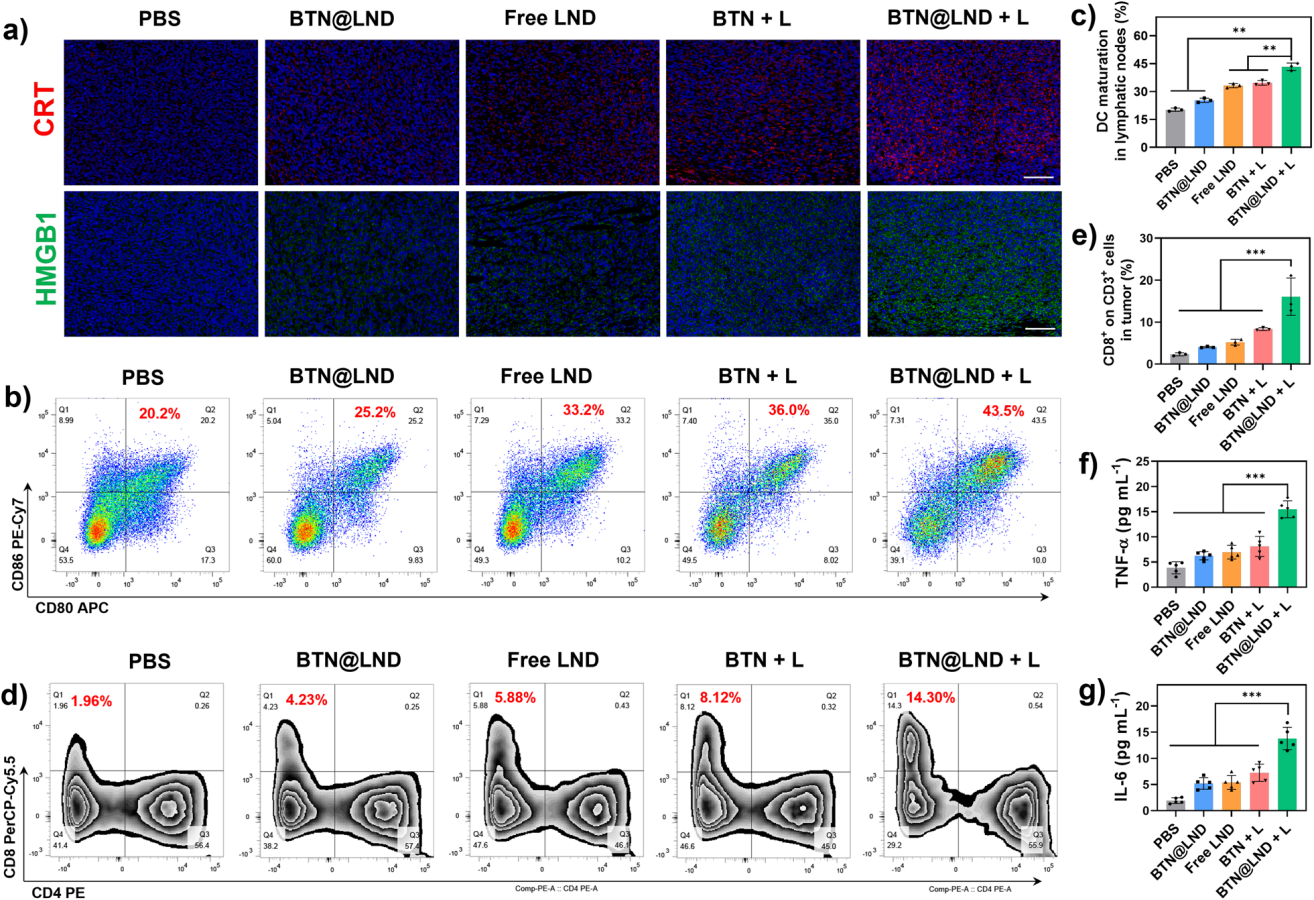
**a** CRT and HMGB1 staining of 4T1 tumor sites after different treatments (scale bar: 100 µm). **b** Representative flow cytometric assay of mature DCs (CD11c^+^CD80^+^CD86^+^) in tumor-draining lymph nodes after various treatments. **c** Quantitative data of mature DCs in lymph nodes corresponding to (**c**) (n = 3). **d** Representative flow cytometric assay of CD4^+^ and CD8^+^ T cells in tumor after various treatments (gated on CD3^+^ T cells). **e** Quantitative data of cytotoxic T cells in tumor corresponding to (d) (n = 3). **f**, **g** Serum levels of TNF-α and IL-6 after different treatments (n = 5). **P < 0.01, ***P < 0.001

To investigate the immune activation of pyroptosis-mediated photoimmunotherapy induced by BTN@LND, principal immunological analyses, including DAMPs release, DC maturation, and cytokine secretion, were performed on the 5th day after treatment with various regimens. The expression levels of CRT and HMGB1 in the tumor sections were evaluated by immunofluorescence staining. The expression levels of HMGB1 and CRT were most prominently upregulated after treatment with BTN@LND + L, thus suggesting the strongest induction of ICD (Fig. 6a). Next, ICD activation-induced immune cell infiltration was analyzed in vivo using flow cytometry. As presented in Fig. 6b, c, the proportion of CD80^+^CD86^+^ DCs in tumor lymphonodus were significantly improved by treatment with BTN@LND + L (43.5%), and this was higher than those of the BTN + L group (36.0%), free LND group (33.2%), BTN@LND group (25.2%), and PBS group (20.2%). To further evaluate the immune response primed by mature DCs, the population and phenotype of tumor-infiltrating lymphocytes were analyzed by detecting their corresponding biomarkers in both the spleen and tumor tissues. Flow cytometry analysis revealed that the highest proportion of effector T cells (CD3^+^CD8^+^) was 25.8% in the spleen after receiving BTN@LND + L treatment (Additional file 1: Figs. S21, S22). Notably, the percentage of T cells at the tumor site increased dramatically to 14.3% after treatment with BTN@LND + L compared to that in the PBS group, thus revealing a 7.3-fold increase (Fig. 6d, e) and indicating the successful activation of antitumor immunity. Additionally, proinflammatory cytokines were detected using an enzyme-linked immunosorbent assay (ELISA). In comparison to the other remedies, the levels of several typical proinflammatory cytokines in the serum of mice in BTN@LND + L group were distinctly elevated, including tumor necrosis factor-α and interleukin-6 (Fig. 6f, g). Overall, these results indicate that the synergistic effect of NIR-II PTT and chemotherapy effectively boosts systemic antitumor immunity.

## Conclusion

In summary, our donor engineering strategy developed a series of D–A–D–type small-molecule NIR-II-absorbing PTAs (termed B2T, BTD, and BTN) and produced an optimal NIR-II-absorbing PTA (BTN) with the greatest NIR-II photothermal performance (ε1064 = 1.51 × 10^4^ M^−1^ cm^−1^, PCE = 75.8%). Moreover, multifunctional liposomes were prepared by co-coating NIR-II PTAs with high NIR-II light harvesting and strong NIR-II photoacoustic capacity into thermally responsive liposomes with an inducer of mitochondrial permeability transition (LND). The liposomes not only achieved NIR-II PAI-guided NIR-II PTT but also improved anti-tumor immunotherapeutic efficacy by significantly inducing tumor cell pyroptosis in the presence of LND, and this enhanced the ICD process and tumor-associated antigen exposure of cancer cells and activated immune effects. This study provides a promising strategy for developing specific pyroptosis/NIR-II PTT synergistic therapies against tumors.

### Supplementary Information

**Additional file 1: Fig. S1–S9.** Nuclear magnetic resonance (NMR) and high-resolution mass spectrometry (HRMS) spectra. **Fig. S10–S22**. The molar extinction coefficient, photothermal curves, stability of nanoparticles, cell uptake, NIR-II photoacoustic imaging, and biocompatibility.

## Data Availability

All data needed to support the conclusions are present in the paper and/or the Supporting Information. Additional data related to this study are available from the corresponding authors upon reasonable.
