# Supplementary material for: A thermoresponsive nanocomposite integrates NIR-II-absorbing small molecule with lonidamine for pyroptosis-promoted synergistic immunotherapy
Source: J Nanobiotechnology. 2024 Apr 10;22:163. doi: 10.1186/s12951-024-02424-5 (PMC11007887; doi:10.1186/s12951-024-02424-5)
Supplement: Supplementary file 1 — Additional file 1: Fig. S1–S9. Nuclear magnetic resonance (NMR) and high-resolution mass spectrometry (HRMS) spectra. Fig. S10–S22. The molar extinction coefficient, photothermal curves, stability of nanoparticles, cell uptake, NIR-II photoacoustic imaging, and biocompatibility. [file 12951_2024_2424_MOESM1_ESM.docx]

**Supporting Information**

**A Thermoresponsive Nanocomposite Integrates NIR-II-absorbing Small Molecule with Lonidamine for Pyroptosis-promoted Synergistic Immunotherapy**

*Pengfei Chen^a^, Chi Zhang^a^, Liuliang He^a^, Mingfei Li^a^, Jie Rong^c^, Pengfei Sun^c^*, Yingying Chen^b^*, Daifeng Li^a^**

*a Department of Orthopedics, The First Affiliated Hospital of Zhengzhou University, Zhengzhou, 450052, China*

*E-mail: lidaifeng@zzu.edu.cn*

*b Department of Gynecology, The First Affiliated Hospital of Zhengzhou University, Zhengzhou, 450052, China*

*E-mail: chenyingying_zzu@163.com*

*c State Key Laboratory of Organic Electronics and Information Displays & Institute of Advanced Materials (IAM), Jiangsu Key Laboratory for Biosensors, Nanjing University of Posts & Telecommunications, Nanjing 210023, China*

*E-mail: iampfsun@njupt.edu.cn*

**Experimental section:**

**Materials.** 4,8-Bis(5-bromo-thiophen-2-yl)-benzo[1,2-*c*;4,5-*c*']bis[1,2,5]thiadiazole) (BBTDT, 97%) was purchased from Alfachem Inc. 2,2-bithiophene (2T), 4,4-bis(6-bromohexyl)-4*H*-cyclopenta[1,2-*b*;3,4-*b*']dithiophene (CPDT), and 4-Hexyl-4H-dithieno[3,2-b:2',3'-d]pyrrole (TBTD). Other catalysts were purchased from commercial sources (such as Aldrich, Energy Chemical and J&K Scientific Ltd.). Unless indicated otherwise, all synthetic procedures were performed in an anhydrous and oxygenfree environment, and all reagents were received from commercial sources. These regents were used without further purification, except toluene which was dried and distilled with N_2_ before use. Cancer cells were obtained from the Shanghai Laboratory Animal Center, Chinese Academy of Science (SLACCAS). The Annexin V-FITC/propidium iodide (PI) cell apoptosis kit and 2-(4-amidinophenyl)-1H-indole-6-carboxamidine (DAPI) were obtained from KeyGen Biotech. Co., Ltd (Nanjing, China). Dulbecco’s Modified Eagle’s Medium (DMEM, Gibco, U.S.) was obtained from Gene Tech Co. (Shanghai, China). The relevant immune kits were purchased from BD Pharmingen.

**Characterization.** The NMR spectra were recorded with a Bruker Ultra Shield Plus 400 MHz spectrometer in deuterated chloroform (CDCl_3_). The molecular analysis was performed on AutoflexIII Matrix-assisted laser desorption/ionization (MALDI) time-of-flight (TOF) mass spectrometry (Bruker Daltonics, USA). The morphology of nanoparticles was determined using a transmission electron microscope (HT7700, TEM) with an acceleration voltage of 100 KV. Dynamic light scattering (DLS) analysis were conducted on a commercial laser light scattering spectrometer (ALV-7004; ALV, Langen, Germany) equipped with a multi-τ digital time correlator and a He-Ne laser (at λ = 632.8 nm). The ⟨*D*_h_⟩ data were extracted through a CONTIN analysis. All samples we used for the test were optically cleared with Millipore filters (0.45 μm). The test was conducted at a 90° scattering angle and room temperature. A Shimadzu UV-3600 spectrophotometer was utilized to record the absorption spectra of our samples at room temperature. All photothermal tests were detected using a Fotric 225 instrument (IR thermal camera, ± 2 °C) purchased from Fotric. (Shanghai, China). The methyl thiazolyl tetrazolium (MTT) and enzyme-linked immunosorbent analysis (ELISA) were conducted using a PowerWave XS/XS2 microplate spectrophotometer (BioTek, Winooski, VT). The flow cytometry experiments were performed using a Flow Sight Imaging Flow Cytometer (Merck Millipore, Darmstadt, Germany).

***In vitro* photothermal effect and photothermal conversion efficiency.** To evaluate the photothermal effect of B2T, BTD, and BTN, three nanoparticle solutions (200 μL) with concentrations of 25, 50, 100, and 200 μg/mL were successively irradiated with 1064 nm laser (1.0 W cm^−2^, 5 min). The temperature changes of the B2T, BTD, and BTN solutions were performed with an IR thermal camera, respectively, and these data were recorded every 30 s.

**Cell Culture.** 4T1 breast cancer cells were cultured with Dulbecco’s Modified Eagle’s Medium (DMEM) supplemented with 10% fetal bovine serum (FBS) in a humidified 5% CO_2_ environment at 37°C.

**Live/Dead Fluorescence Assays.** 4T1 cells (1 × 10^5^) were grown on slices in a 24-well culture plate for 12 h. Materials in different groups (PBS, BTN@LND, Free LND, BTN + L, and BTN@LND + L) were added to fresh DMEM to obtain the mixed medium (0.1 mg/mL) for 12 h with or without laser irradiation at1064 nm (1.0 W/cm for 5 min). Five hours after the treatment, the cells were costained with calcein-AM and propidium iodide for 30 min. Finally, cells were washed with PBS twice and imaged by CLSM. (Calcein-AM, Ex/Em: 488/510 nm; PI, Ex/Em: 530/615 nm).

**Animal Experiments.** The animal protocols used in this study were approved by the Institutional Committee on the Ethics of Animal Experiments of Zhengzhou University, Zhengzhou, China. All animal procedures were performed in compliance with the Guide for the Care and Use of Laboratory Animals from the National Institutes of Health.

***In Vivo* NIR-II Photoacoustic Imaging.** When the tumors reached a volume of 80-100 mm^3^, 4T1 tumor bearing model mice were intravenously injected with BTN@LND (1.5 mg/mL, 150 μL). Mice are mounted on specially designed holders, which rotate 360 degrees with sophisticated rotating motors. Four short pulsed laser beams (biorthogonal, bioblique) are emitted by a laser the tissue absorbs light energy to generate ultrasonic signal, which is received by a professionally designed 125° are transducer. The signal was collected at 360°, and the photoacoustic image was reconstructed by special 3D reconstruction software through unit transformation of DAQ data.

***In Vivo* NIR-II Photothermal Therapy.** When the tumor volume reached 80-100 mm^3^, the 4T1 tumor-bearing mice were weighed, randomly divided into different treatment groups. About 24 h after the intravenous injection, the mice were divided into groups according to the corresponding treatment methods (n = 5 per group). Tumor volume and mice weight were assessed every other day in the next 12 days. After 12 days, these mice were sacrificed, and pathological analysis of tumors and major organs was performed. The histological tumor sections were observed using an optical microscope. The weight of the tumors were also measured.

**Histological Analysis.** All major organs (heart, liver, spleen, lung, and kidney) and tumor were achieved from healthy ICR mice (n = 3 per group) at 12 days. They were embedded in an optimal cutting temperature sample (Tissue-Tek, Sakura Finetek, USA). After that, these samples were sliced into 4 μm sections with a microtome in the cryostat at -20°C and then transferred to a microscope slide for analysis of hematoxylin-eosin (H&E).

**Graphpad Prism was used for statistical analysis.** Quantitative data were performed for at least three times and the data were expressed as mean ± standard deviation (SD). Data were analyzed for statistical significance using Student’s t-test. **P* < 0.05 was considered statistically significant, while ***P* < 0.01 and ****P* < 0.001 were considered highly and extremely significant.

**Synthesis and Characterization.**

**
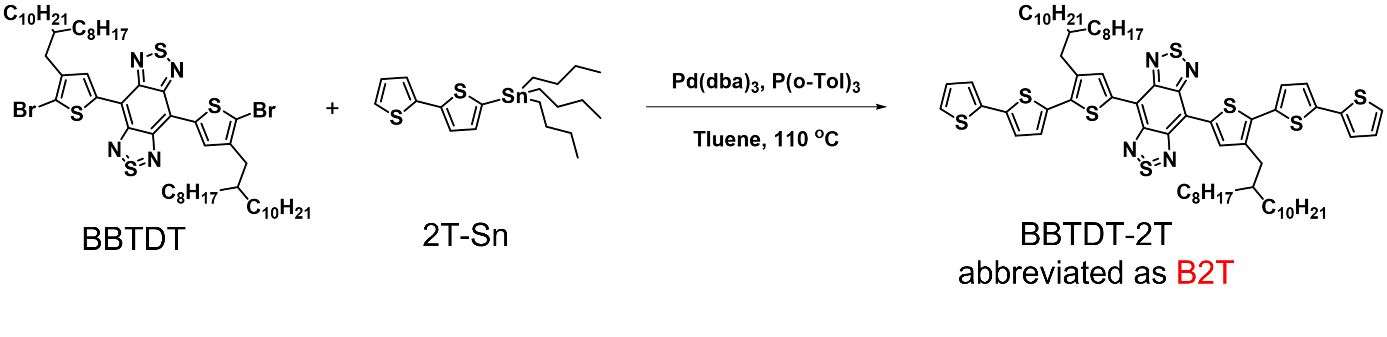
**

**Scheme S1.** The synthetic route of **B2T**.

**Synthesis of B2T.** The solution of **BBTDT** (100 mg, 93 μmol), **2T-Sn** (105 mg, 230 mmol), Pd_2_(dba)_3_ (10 mg) and *trio*-tolylphosphine (32 mg) in anhydrous toluene (10 mL) was stirred at 110 °C for 12 h under N_2_ protection. After cooling, the resulting product was evaporated and purified using column chromatography (hexane: dichloromethane = 4:1) to afford the product **B2T** (86 mg, yield: 74%).

^1^H NMR (400 MHz, CDCl_3_) *δ*: 0.84-0.89 (m, 12H), 1.23-1.31 (m, 48H), 1.35-1.40 (m, 16H), 1.89 (s, 2H), 2.86 (d, 4H), 7.07 (t, 2H), 7.18 (d, 2H), 7.25-7.27 (m, 6H), 8.75 (s, 2H). ^13^C NMR (100 MHz, CDCl_3_) *δ*: 14.15, 22.71, 26.54, 29.41, 29.47, 29.74, 29.81, 29.84, 30.24, 31.96, 31.99, 33.55, 34.18, 38.50, 112.39, 123.66, 124.13, 124.50, 127.93, 135.52, 136.86, 137.27, 151.01. MALDI-TOF: calculated C_70_H_94_N_4_S_8_ (m/z): 1246.5244, found 1247.1150.


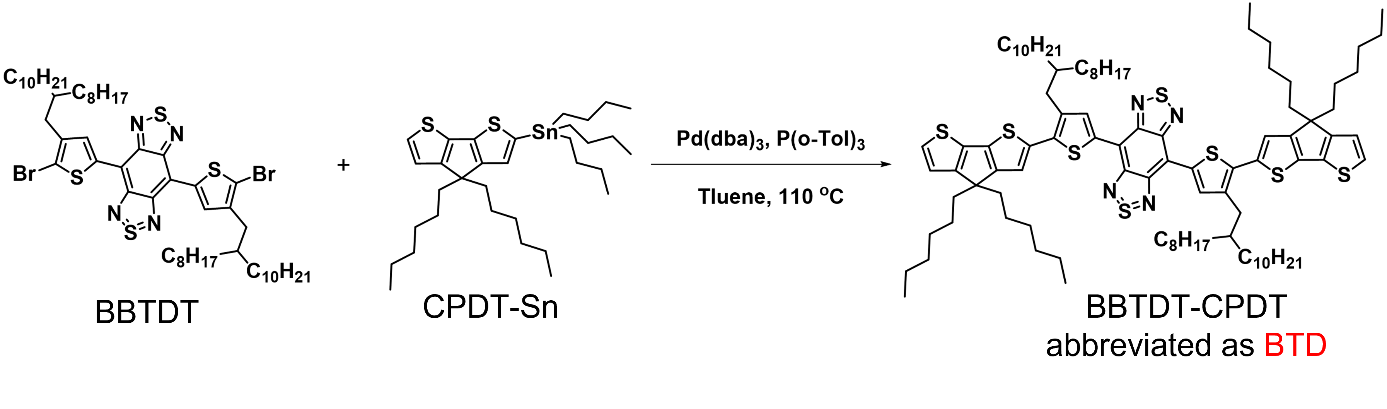


**Scheme S2.** The synthetic route of BTD.

**Synthesis of BTD.** The synthesis method of **BTD** was similar to that of **B2T**, and the yield was 65%.

^1^H NMR (400 MHz, CDCl_3_) *δ*: 0.83 (t, 12H), 0.85 (t, 12H), 1.18-1.29 (m, 82H), 1.33-1.40 (m, 16H), 1.90 (t, 8H), 2.29 (s, 4H), 6.97 (s, 2H), 7.17-7.24 (m, 4H), 8.90 (s, 2H). ^13^C NMR (100 MHz, CDCl_3_) *δ*: 14.10, 14.15, 22.70, 22.71, 24.61, 26.51, 29.40, 29.76, 29.80, 30.20, 31.71, 31.95, 31.97, 112.51, 118.86, 119.04, 120.82, 121.71, 123.50, 124.39, 125.41, 135.19, 136.22, 139.19, 151.36, 158.32, 158 .64. MALDI-TOF: calculated C_96_H_142_N_4_S_8_ (m/z): 1607.9034, found 1609.8156.


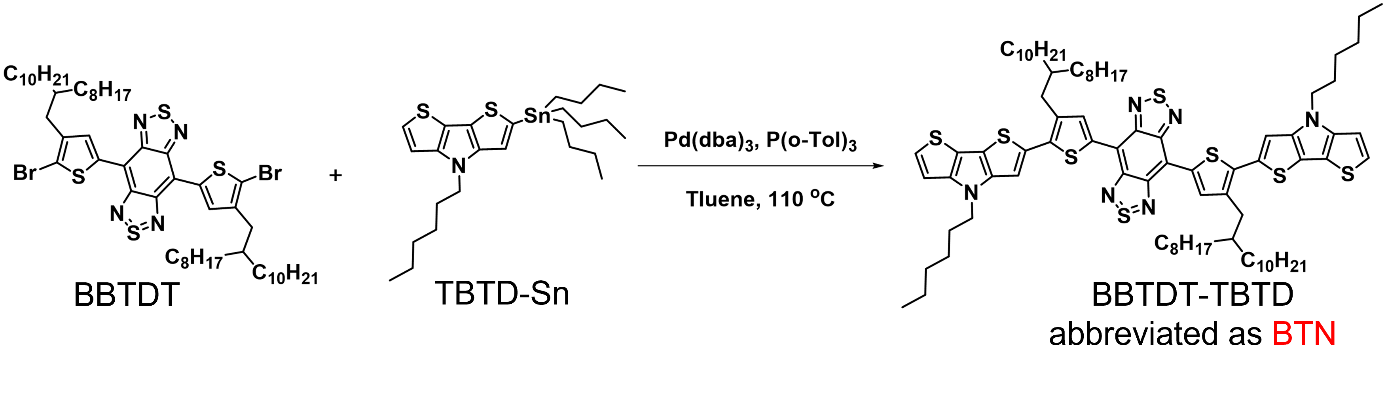


**Scheme S3.** The synthetic route of **BTN**.

**Synthesis of BTN.** The synthesis method of **BTN** was similar to that of **B2T**, and the yield was 69%.

^1^H NMR (400 MHz, CDCl_3_) *δ*: 0.83 (t, 6H), 0.85 (t, 6H), 0.90 (t, 6H), 1.20-1.25 (m, 52H), 1.32-1.40 (m, 24H), 1.88-1.95 (m, 6H), 2.95 (s, 4H), 4.22 (t, 4H), 7.02 (s, 2H), 7.18 (s, 2H), 7.29 (s, 2H), 8.89 (s, 2H). ^13^C NMR (100 MHz, CDCl_3_) *δ*: 14.10, 14.15, 22.62, 22.72, 26.52, 26.80, 29.41, 29.49, 29.74, 29.81, 29.83, 29.85, 30.28, 30.44, 31.53, 31.96, 31.98, 33.51, 34.34, 38.51, 47.40, 109.55, 110.89, 112.17, 114.91, 115.73, 123.65, 133.87, 135.32, 137.04, 139.09, 144.92, 145.21, 151.09. MALDI-TOF: calculated C_82_H_116_N_6_S_8_ (m/z): 1440.7127, found 1442.4466.


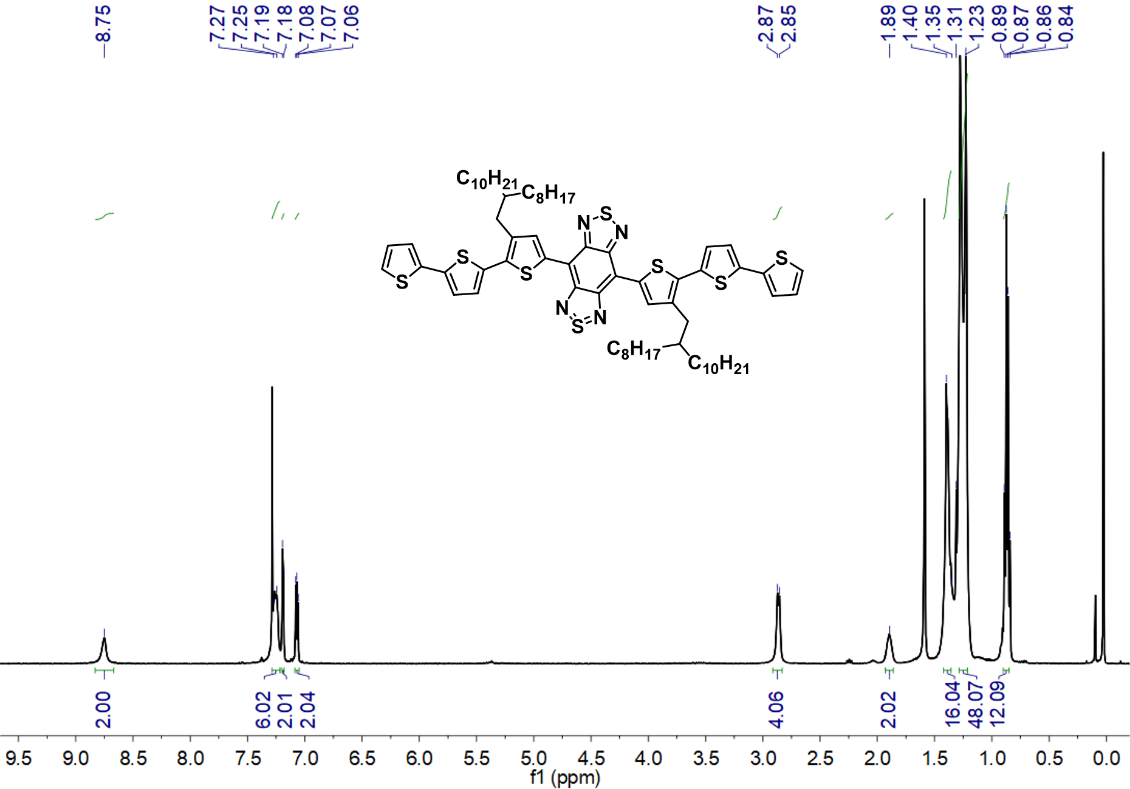


**Figure S1.** ^1^H NMR spectrum of **B2T** in CDCl_3_.


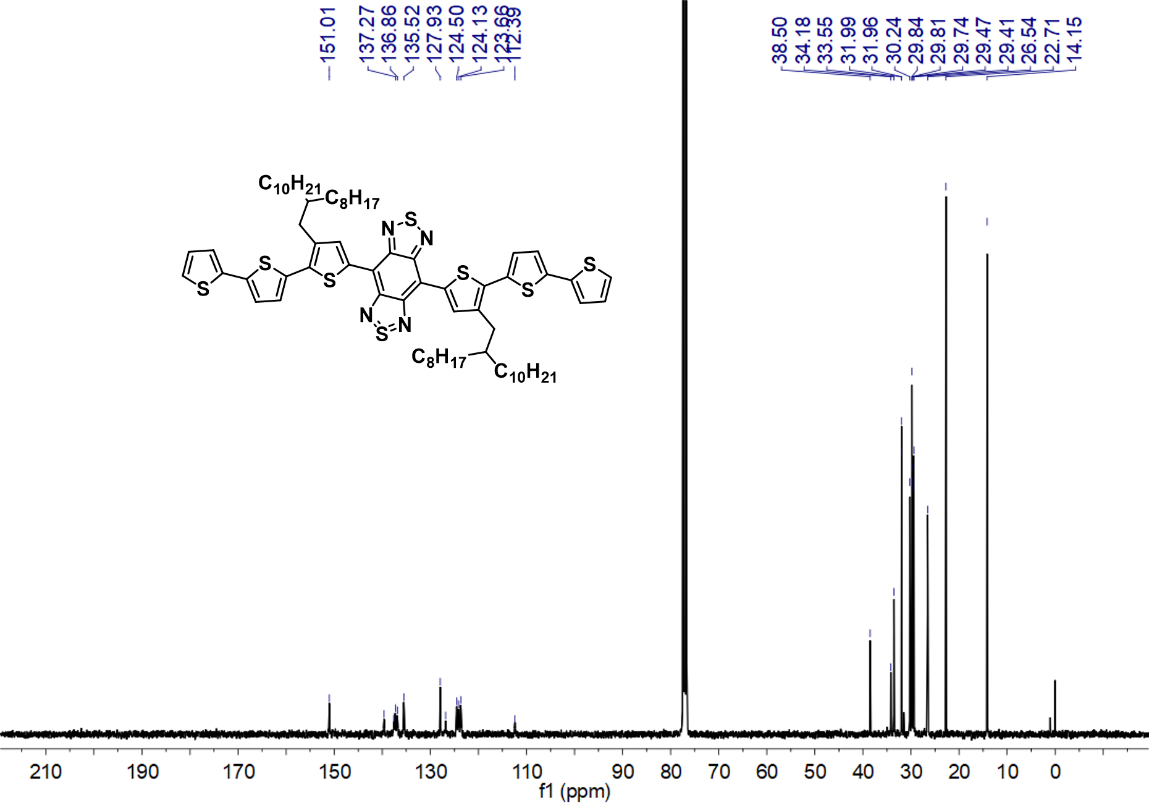


**Figure S2.** ^13^C NMR spectrum of **B2T** in CDCl_3_.


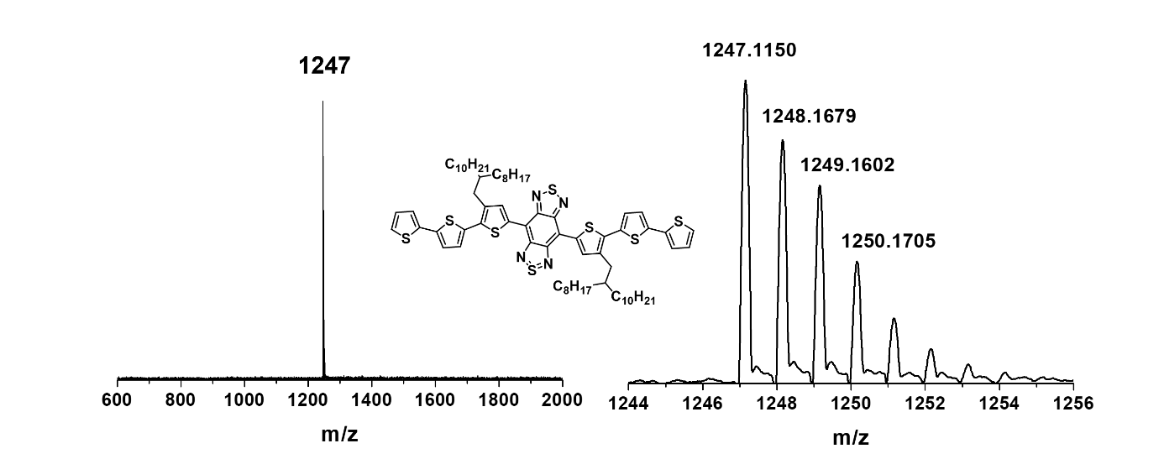


**Figure S3.** MALDI-TOF mass spectrometry of **B2T**.


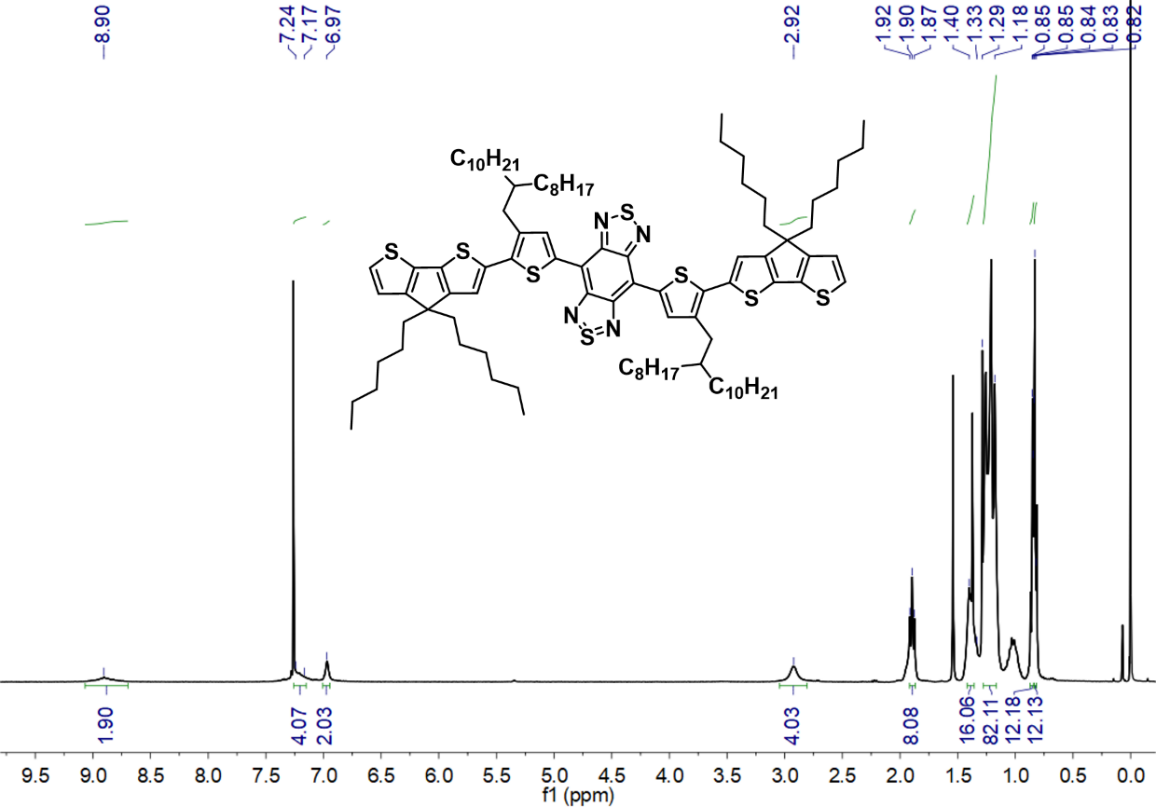


**Figure S4.** ^1^H NMR spectrum of **BTD** in CDCl_3_.

**
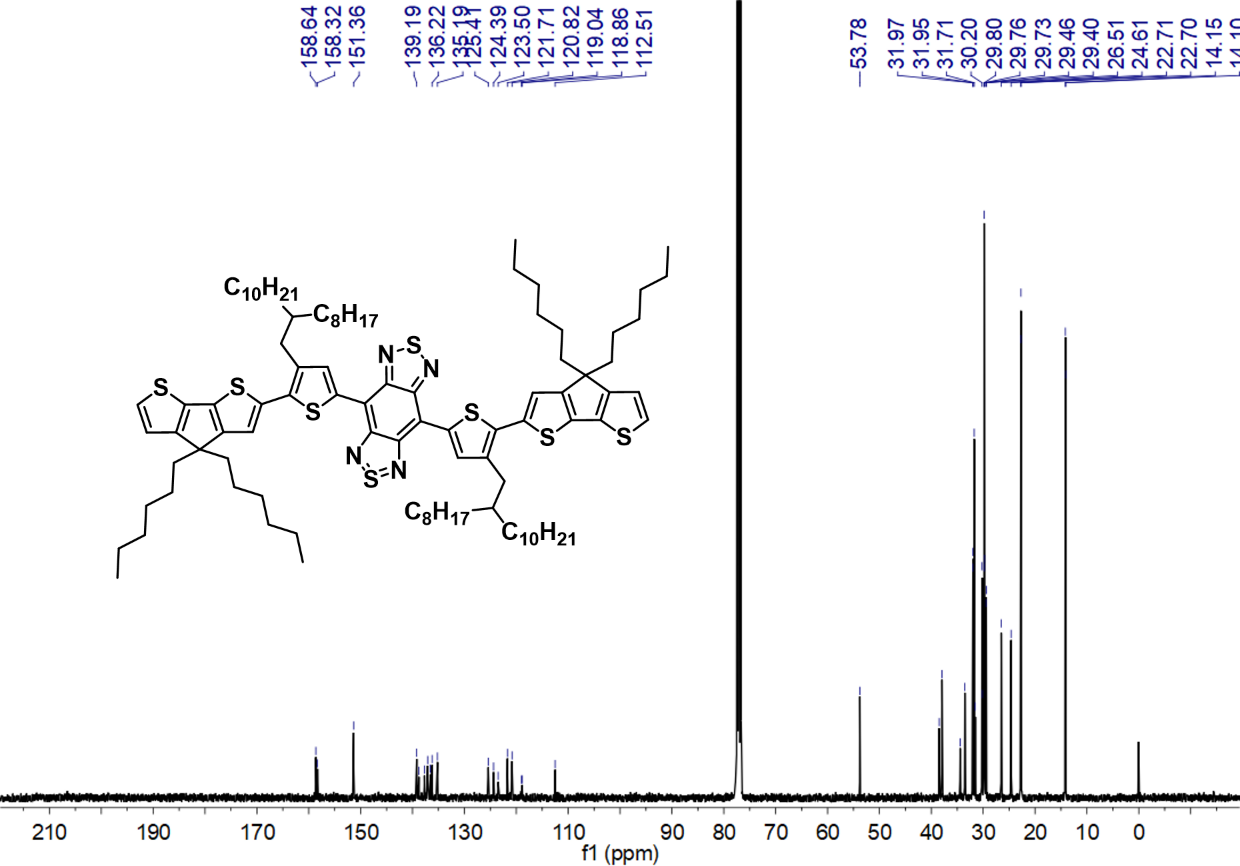
**

**Figure S5.** ^13^C NMR spectrum of **BTD** in CDCl_3_.


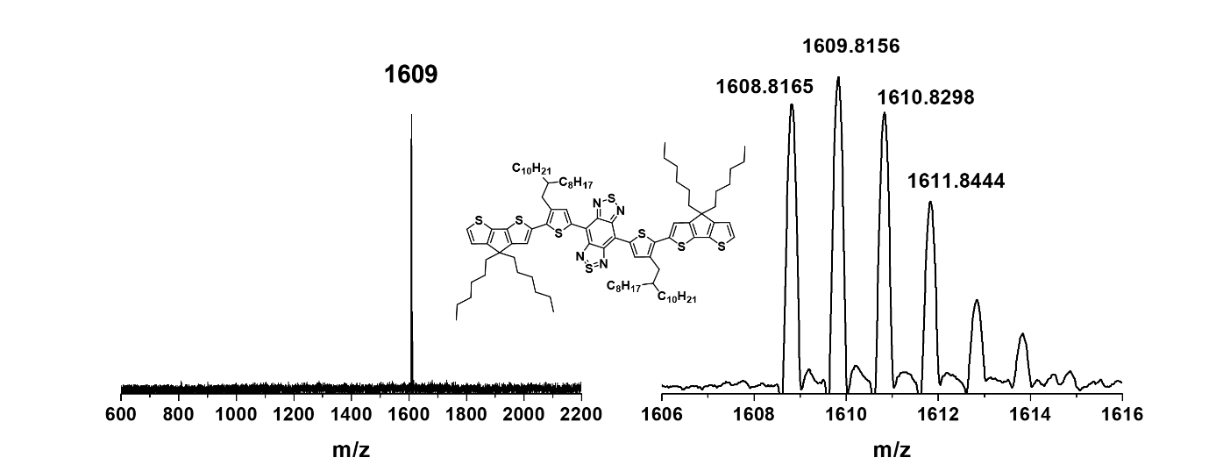


**Figure S6.** MALDI-TOF mass spectrometry of **BTD**.


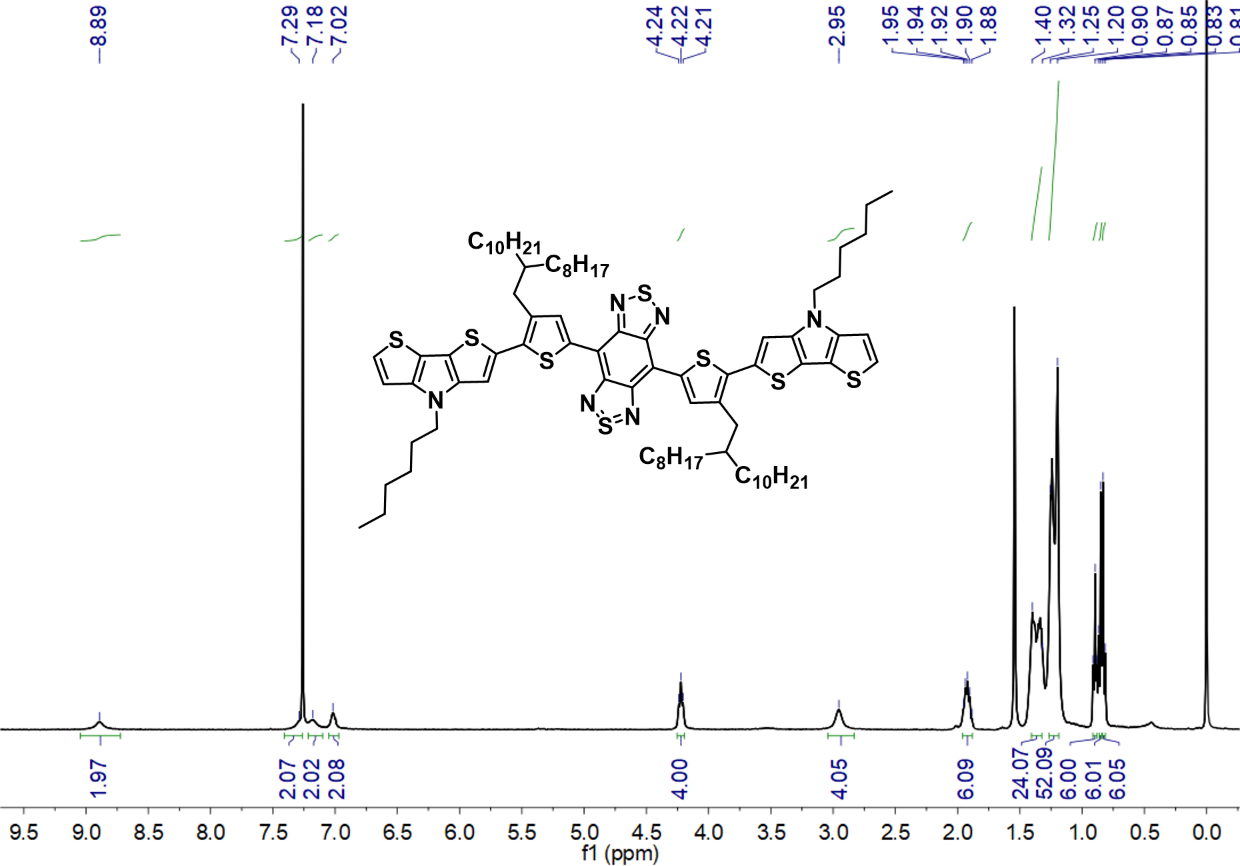


**Figure S7.** ^1^H NMR spectrum of **BTN** in CDCl_3_.


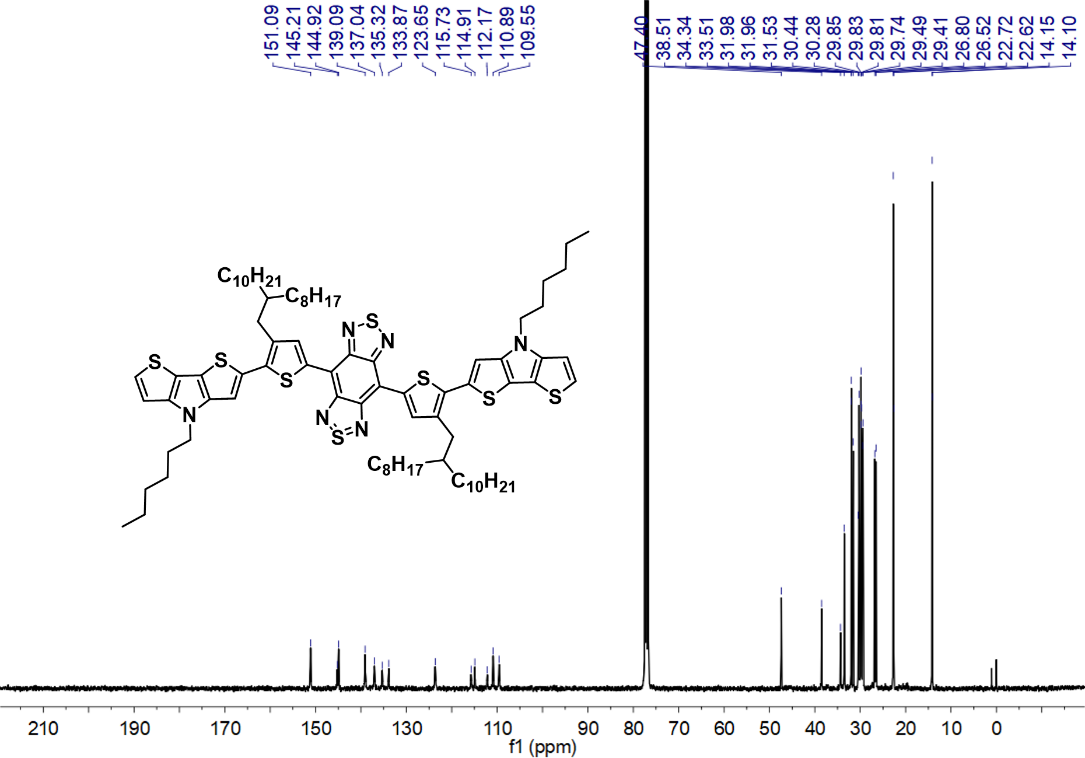


**Figure S8.** ^13^C NMR spectrum of **BTN** in CDCl_3_.


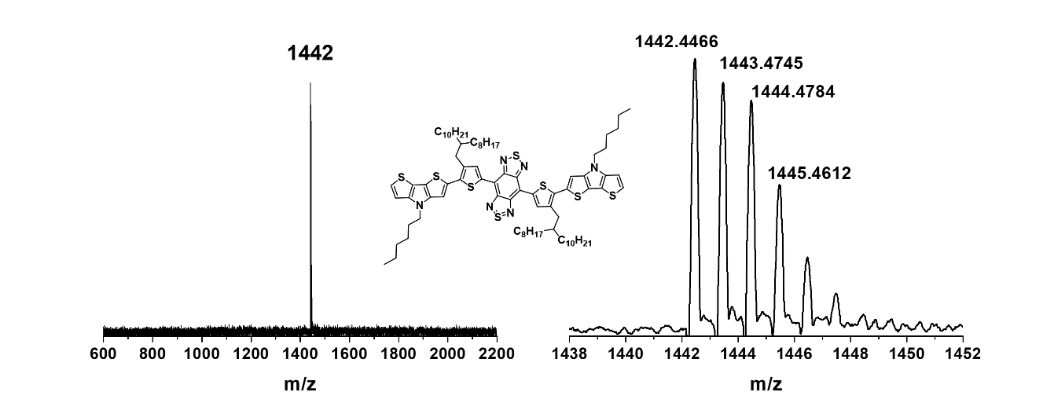


**Figure S9.** MALDI-TOF mass spectrometry of **BTN**.


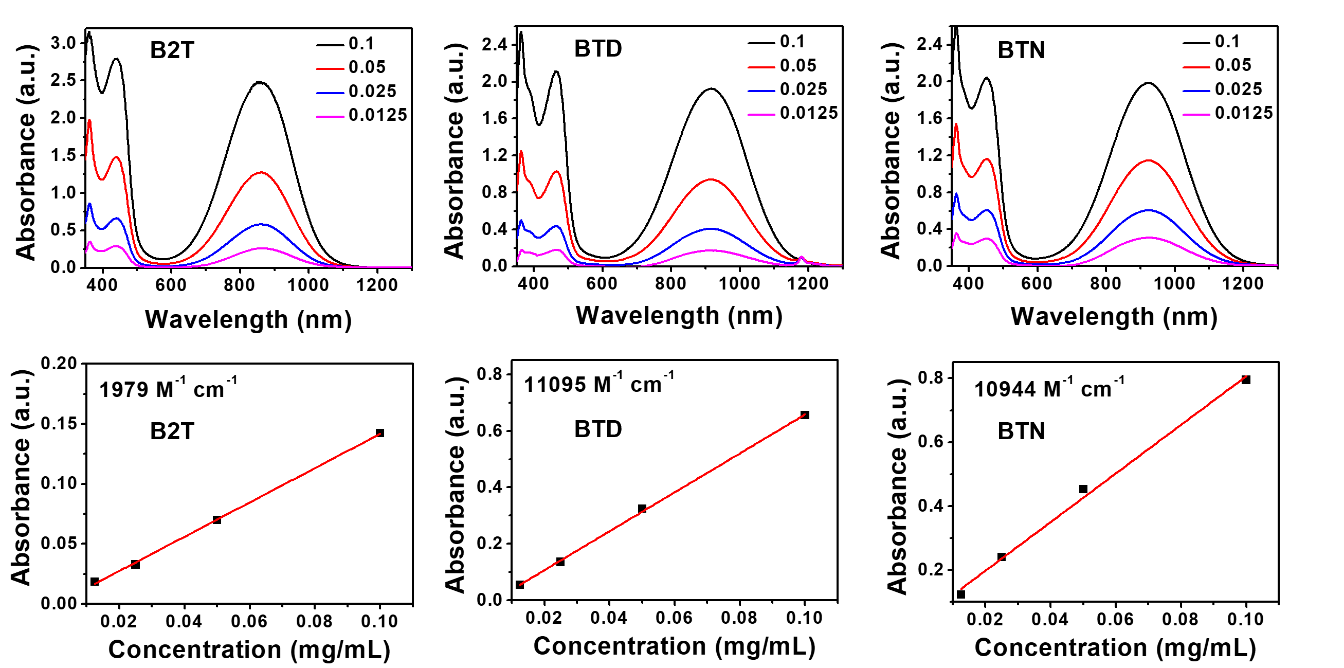


**Figure S10.** The molar extinction coefficient of B2T, BTD, and BTN in THF at 1064 nm.


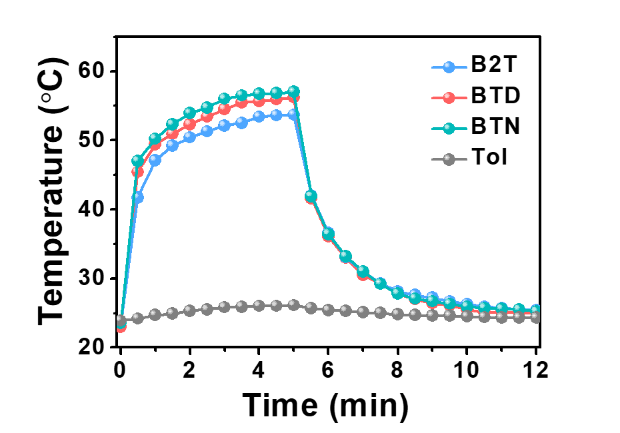


**Figure S11.** Photothermal curves in toluene under laser (808 nm, 0.60 W cm^−2^) irradiation.


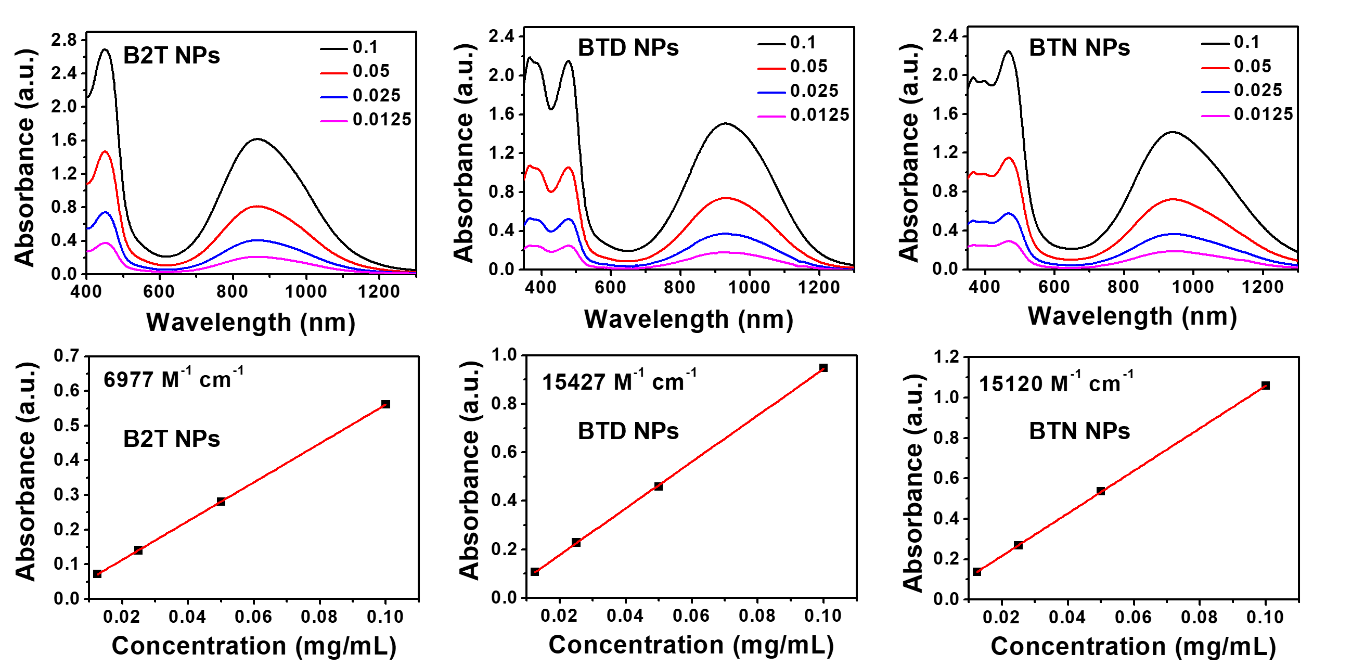


**Figure S12.** The molar extinction coefficient of B2T NPs, BTD NPs, and BTN NPs in water at 1064 nm.


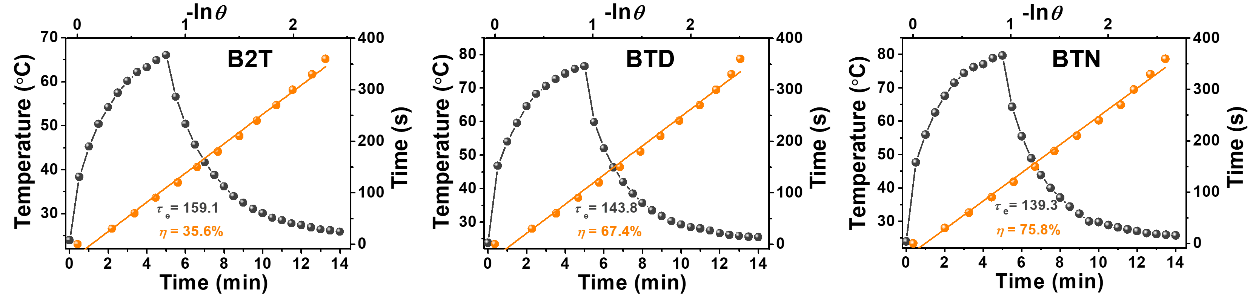


**Figure S13.** Linear correlation of the cooling times versus negative natural logarithm of driving force temperatures in water, respectively.


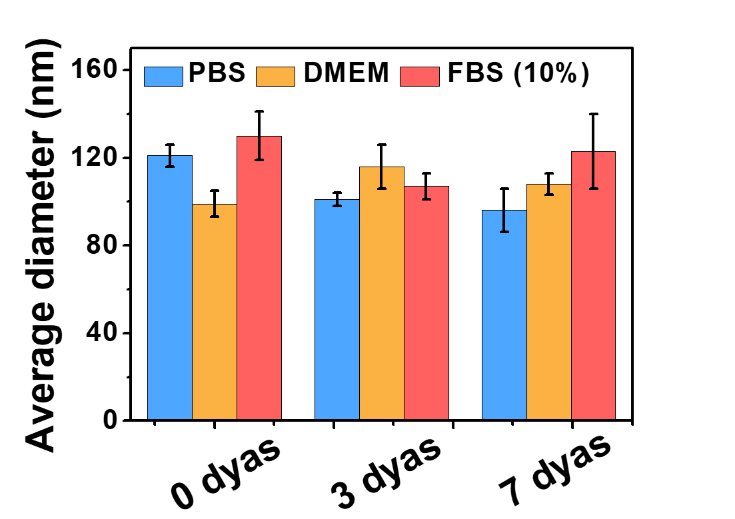


**Figure S14.** Average diameters of BTN@LND NPs in PBS, DMEM, or FBS for different time periods.


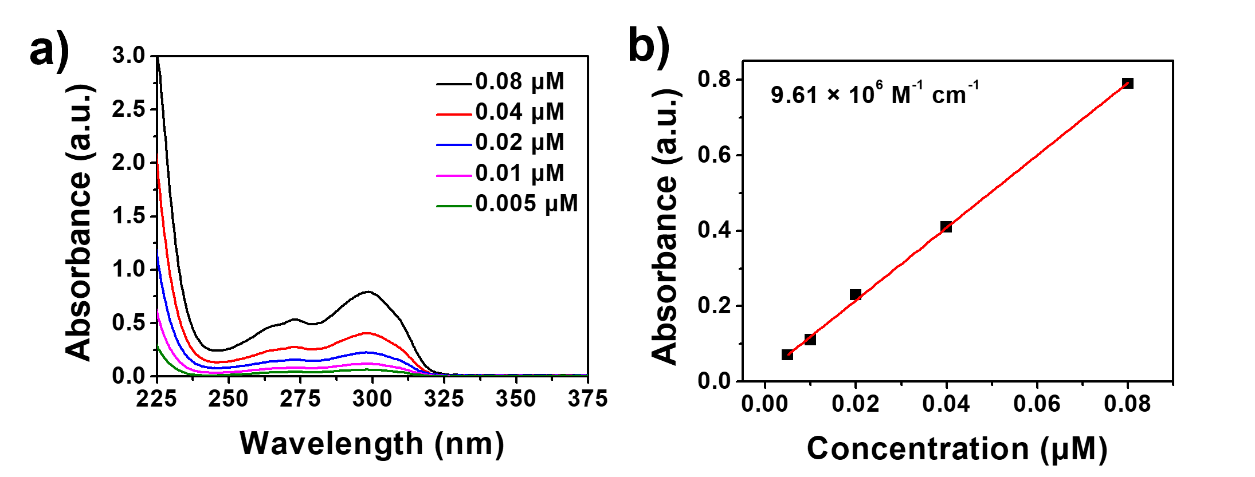


**Figure S15.** The molar extinction coefficient of LND in water at 298 nm.


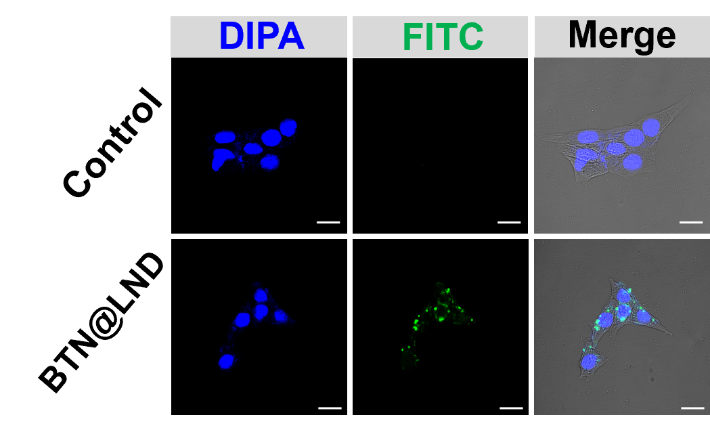


**Figure S16.** Cell uptake ability of BTN@LND NPs. Fluorescence imaging of 4T1 cells after co-staining with FITC@ BTN@LND NPs (green) and DAPI (blue). Scale bar, 20 μm.


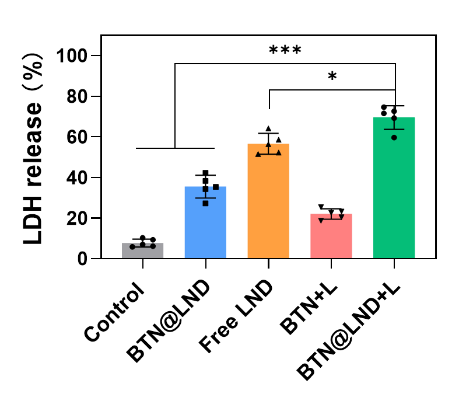


**Figure S17.** g) LDH release of 4T1 cells receiving different treatments (n = 5).


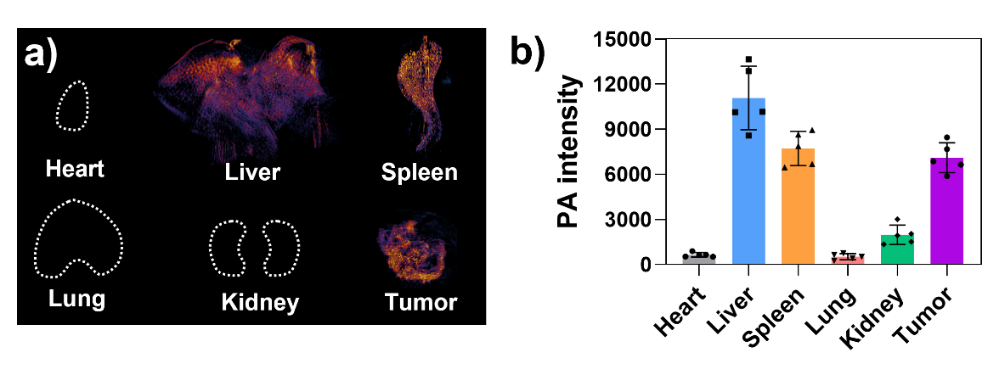


**Figure S18.** a) *Ex vivo* NIR-II photoacoustic imaging of the tumor and main organs of BTFQ/DMPC after 36 h intravenous administration. b) corresponding signal quantification. Error bars, mean ± SD (n = 5).


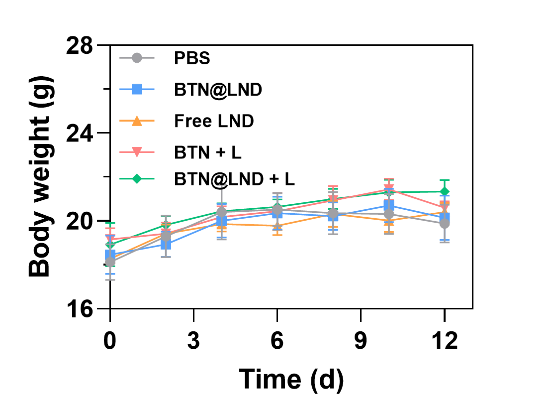


**Figure S19.** Body-weight growth curves of different treatment. Error bars, mean ± SD (n = 5).


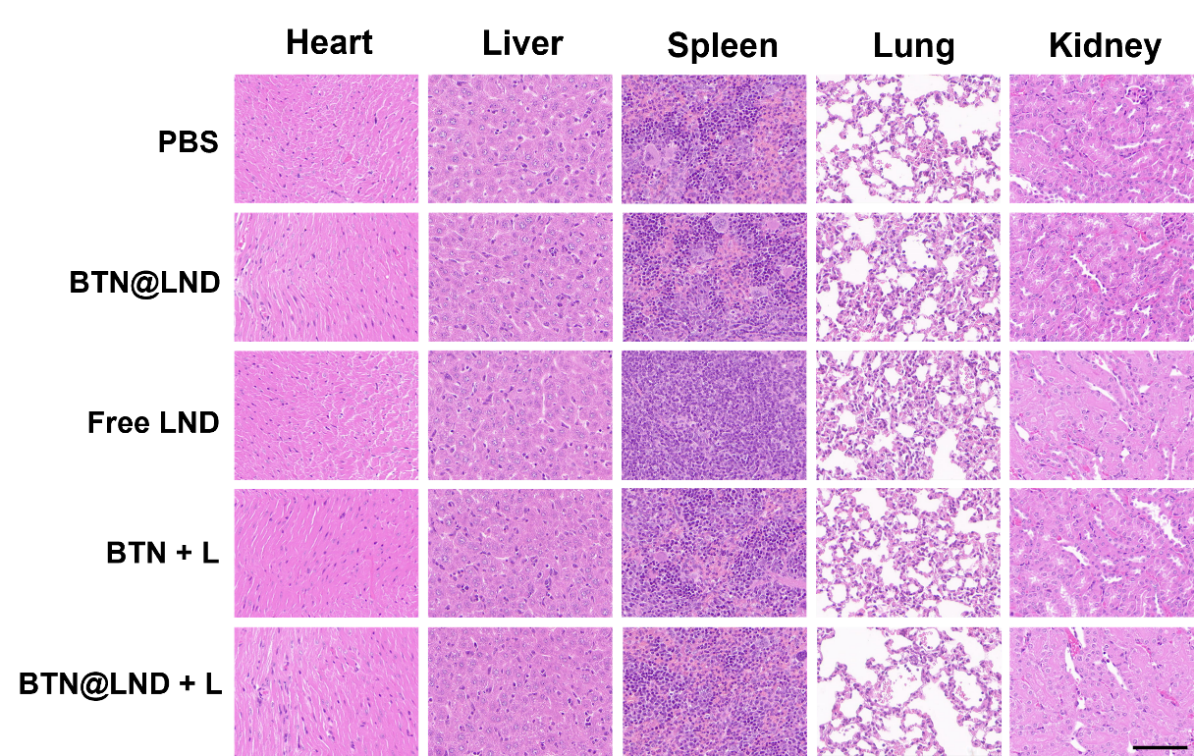


**Figure S20.** Images of H&E-stained main mouse organs of BTN@LND + L treatment after 12 days. scale bars, 100 μm.


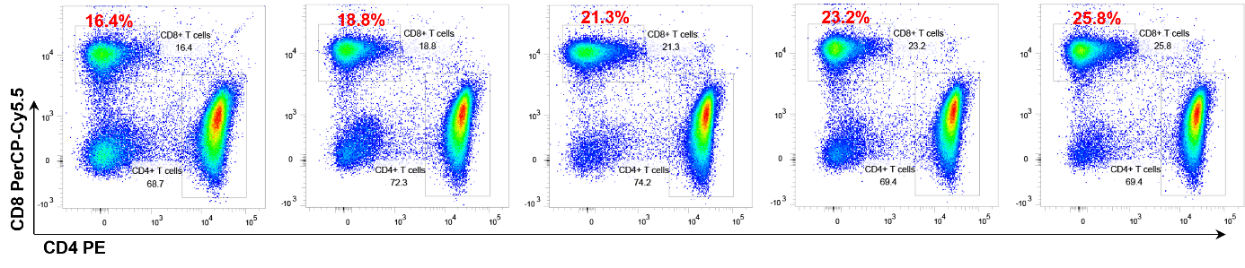


**Figure S21.** Representative flow cytometric assay of CD4^+^ CD8^+^ T cells in spleen after various treatments.


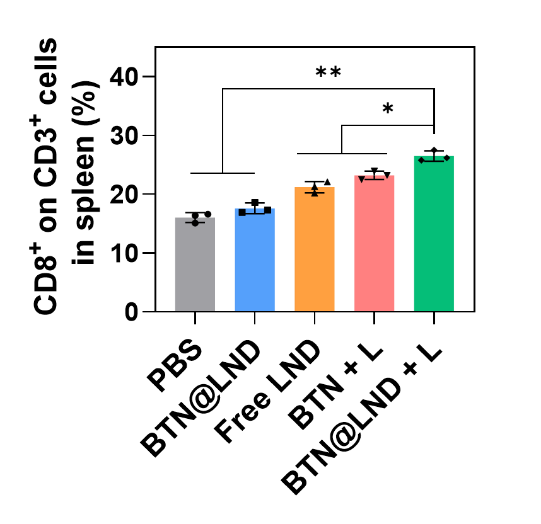


**Figure S22.** Quantitative data of cytotoxic T cells in spleen after various treatments (n = 3).
